# Molecular MRI Reveals Macrophage‐Enriched Peritumoral Immune Remodeling After Incomplete Ablation in Liver Cancer

**DOI:** 10.1002/advs.78490

**Published:** 2026-11-05

**Authors:** Yubei He, Han Xu, Julian M. M. Rogasch, Nikolaus Berndt, Jing Guo, Eyk Schellenberger, Akvile Häckel, Luisa Heidemann, Friederike Hesse, Yu Liu, Justus Ramtke, Nicola Beindorff, Frank Hofheinz, Avan Kader, Michael Mülleder, Kathrin Textoris‐Taube, Bernd Hamm, Dominik N. Müller, Robin Schmidt, Ralf Hauptmann, Heike Traub, Mathias Schannor, Lynn Jeanette Savic

**Affiliations:** ^1^ Department of Radiology Charité – Universitätsmedizin Berlin Corporate Member of Freie Universität Berlin and Humboldt – Universität zu Berlin Berlin Germany; ^2^ Experimental and Clinical Research Center (ECRC) A Cooperation of Charité – Universitätsmedizin Berlin and Max Delbrück Center for Molecular Medicine (MDC) Berlin Germany; ^3^ Department of Nuclear Medicine Campus Virchow‐Klinikum Charité‐Universitätsmedizin Berlin Berlin Germany; ^4^ Department of Molecular Toxicology German Institute of Human Nutrition Potsdam – Rehbruecke (DIfE) Nuthetal Germany; ^5^ Berlin Institute of Health at Charité – Universitätsmedizin Berlin Berlin Germany; ^6^ Berlin Experimental Radionuclide Imaging Center (BERIC, RRID: SCR_028573) Charité Universitätsmedizin Berlin, Corporate Member of Freie Universität Berlin and Humboldt Universität zu Berlin Berlin Germany; ^7^ Helmholtz – Zentrum Dresden – Rossendorf, PET Center Institute of Radiopharmaceutical Cancer Research Dresden Germany; ^8^ Department of Diagnostic and Interventional Radiology Technical University of Munich Munich Germany; ^9^ Core Facility High – Throughput Mass Spectrometry Charité – Universitätsmedizin Berlin Freie Universität Berlin and Humboldt – Universität zu Berlin Berlin Germany; ^10^ Charité – Universitätsmedizin Berlin Corporate Member of Freie Universität Berlin and Humboldt – Universität zu Berlin Berlin Germany; ^11^ Max Delbrück Center for Molecular Medicine in the Helmholtz Association Berlin Germany; ^12^ Bundesanstalt für Materialforschung und – prüfung (BAM) Berlin Germany; ^13^ Freie Universität Berlin Institute of Geological Sciences Berlin Germany; ^14^ Department of Radiology and Nuclear Medicine Otto – von – Guericke University Magdeburg Magdeburg Germany

**Keywords:** europium‐doped very small superparamagnetic iron oxide nanoparticles, liver cancer, macrophages‐associated remodeling, microwave ablation, multiparametric MRI, tumor microenvironment

## Abstract

Incomplete image‐guided ablation can promote local tumor progression by reshaping the residual tumor microenvironment toward immunosuppression, yet noninvasive biomarkers of this process remain limited. Here, a translational multiparametric PET/MRI strategy was developed to monitor macrophage‐associated microenvironmental remodeling after incomplete microwave ablation (iMWA) in orthotopic VX2 liver tumors. Twenty‐four tumor‐bearing rabbits were assigned to untreated control, early post‐iMWA (short‐term), or late post‐iMWA (long‐term) groups and underwent clinical 3 T PET/MRI, including diffusion‐weighted imaging, dynamic contrast‐enhanced MRI, [^1^ ^8^F]FDG PET, and T2‐weighted MRI before and after administration of Eu‐VSOP. Eu‐VSOP‐enhanced MRI revealed a hypointense peritumoral rim after iMWA, with the largest rim‐to‐liver signal decrease and rim thickness observed in the long‐term group. Prussian blue staining and LA‐ICP‐ToF‐MS demonstrated preferential iron and europium enrichment in macrophage‐rich, CD206‐associated peritumoral regions. Histology and proteomics further demonstrated increased CD206 and MMP‐9 expression, extracellular matrix remodeling, immunosuppressive myeloid‐associated changes, and metabolic reprogramming in viable residual tumors, accompanied by altered [^1^ ^8^F]FDG uptake. Together, these findings indicate that incomplete ablation induces a temporally evolving, macrophage‐rich immunosuppressive peritumoral niche and support the potential of Eu‐VSOP‐enhanced multiparametric PET/MRI for noninvasive monitoring of post‐ablation microenvironmental remodeling.

## Graphical abstract

Incomplete microwave ablation (iMWA) induced a spatially organized, macrophage‐rich immunosuppressive tumor microenvironment (TME) in orthotopic VX2 liver tumors. Molecular MRI enhanced with europium‐doped very small superparamagnetic iron oxide nanoparticles (Eu‐VSOP) noninvasively visualized this remodeling, showing preferential nanoparticle enrichment in macrophage‐rich, CD206‐associated peritumoral regions. This approach may provide a translational imaging strategy to complement conventional assessment by identifying biologically adverse post‐ablation microenvironments and informing risk stratification following incomplete liver tumor ablation.

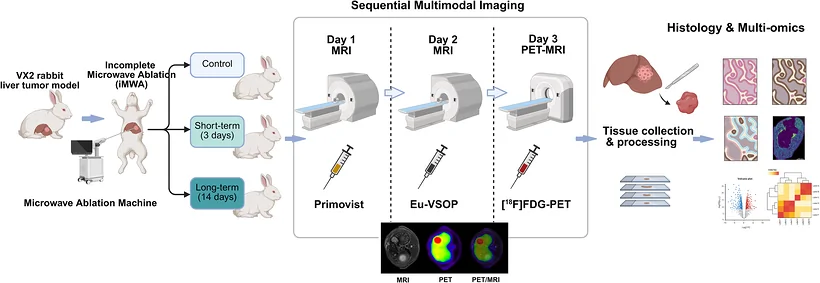

**Abbreviations**
ADC: Apparent Diffusion Coefficient
DCE/MRI: Dynamic Contrast‐enhanced MRI
DWI: Diffusion‐weighted Imaging
Eu‐VSOP: Europium‐doped very small superparamagnetic iron oxide nanoparticles
GO‐BP: Gene ontology biological process
GSEA: Gene Set Enrichment Analysis
H&E: Hematoxylin and Eosin
HCC: Hepatocellular carcinoma
ICCs: Intraclass Correlation Coefficients
iMWA: incomplete Microwave Ablation
LA‐ICP‐ToF‐MS: Laser Ablation in combination with Inductively Coupled Plasma Time‐of‐Flight Mass Spectrometry
MDSCs: Myeloid‐derived Suppressor Cells
PCA: Principal component analysis
PET/MRI: Positron Emission Tomography / Magnetic Resonance Imaging
PPI: Protein–protein Interaction
ROIs: Regions of Interest
SUV: Standardized Uptake Values
TAMs: Tumor‐Associated Macrophages
TME: Tumor Microenvironment

## 1 Introduction

Liver cancer is the sixth most commonly diagnosed malignancy worldwide and the third leading cause of cancer‐related death [1, 2]. Thermal ablation is a guideline‐recommended treatment that is widely used in patients with early‐ to intermediate‐stage hepatocellular carcinoma (HCC) and liver metastases [3, 4]. Among thermal ablation techniques, microwave ablation offers advantages over radiofrequency ablation by enabling more rapid and deeper tissue heating, making it particularly effective for treating larger lesions [5].

Despite its efficacy, local recurrence after ablation remains a major challenge in the management of liver malignancies [6, 7]. Incomplete ablation can expose surviving tumor cells to sublethal heat stress, potentially enhancing their aggressiveness [8]. Preclinical studies show that such heat stress activates oncogenic pathways such as PI3K/mTOR/AKT and metabolic reprogramming via O‐GlcNAcylation, promoting tumor proliferation and recurrence [9, 10]. In colorectal cancer liver metastases, incomplete RFA was shown to suppress T‐cell activity and impair PD‐1 checkpoint inhibitor therapy, accelerating tumor growth [11]. Furthermore, ablation‐induced remodeling of the immune microenvironment by altered cytokine or chemokine expression in residual tumor cells may contribute to tumor immune evasion and resistance [12, 13].

In addition to myeloid‐derived suppressor cells (MDSCs) and regulatory T cells, tumor‐associated macrophages (TAMs) are key components of the tumor microenvironment (TME) that contribute to tumor immune evasion [14, 15]. Although TAM phenotypes are understood to span a continuum of activation states rather than a strict binary classification, the M1‐like (classically activated) and M2‐like (alternatively activated) programs remain useful references to describe predominant functional tendencies in the TME [16]. Briefly, M1‐like macrophages are generally associated with pro‐inflammatory and antitumor functions [17], whereas M2‐like macrophages are linked to immunosuppression, tissue remodeling, angiogenesis, and tumor progression [18]. Accordingly, noninvasive detection of macrophage‐rich immunosuppressive remodeling may provide complementary information beyond conventional assessment of tumor viability and morphology.

Current post‐ablation imaging primarily relies on morphological and contrast‐enhanced criteria to identify residual viable tumors. However, these approaches provide limited information regarding treatment‐induced immune and stromal remodeling. Molecular imaging strategies may therefore complement these conventional imaging techniques by enabling noninvasive assessment of biological changes within the post‐ablation TME [19].

Thus, this translational study aimed to investigate whether iMWA induces a spatially organized, macrophage‐associated immunosuppressive TME in liver tumors, and whether this process can be noninvasively visualized using molecular imaging. Eu‐VSOP‐enhanced MRI was used to assess peritumoral tissue regions associated with macrophage‐rich remodeling, with europium doping enabling ex vivo elemental validation. Utilizing iMWA in the orthotopic VX2 rabbit liver tumor model, longitudinal Eu‐VSOP‐enhanced molecular imaging findings were integrated with histopathologic and proteomic biomarkers to evaluate its potential for noninvasive visualization of post‐ablation immunometabolic remodeling and risk stratification following incomplete liver tumor ablation.

## 2 Materials and Methods

### 2.1 Translational Study Design

Thirty New Zealand white rabbits were used in this study, including six donor rabbits and 24 experimental VX2 liver tumor‐bearing rabbits. The 24 tumor‐bearing rabbits were randomized into one control and two treatment groups (n = 8 per group): (1) Control group – received no treatment and underwent multiparametric (mp) PET/MRI after 2–3 weeks of tumor growth; (2) short‐term group‐ underwent iMWA targeting at least 50% of the tumor, and mpPET/MRI 1–3 days post‐iMWA; (3) long‐term group‐ underwent multiparametric PET/MRI 14–17 days post‐iMWA. Rabbits in both treatment groups had baseline scans to confirm tumor presence and viability prior to iMWA. For all groups, experimental imaging was performed over three days using different contrast media: Day 1 – gadolinium‐enhanced MRI for vascularization and viability; Day 2 – MRI was performed before and after Eu‐VSOP injection; Day 3 – Eu‐VSOP‐enhanced MRI was acquired 24 h after Eu‐VSOP injection, together with [^18^F]FDG‐PET/MRI to assess tumor metabolic activity. After final imaging, rabbits were euthanized, and tissue was collected for ex vivo analyses. Signal alterations on T2‐weighted MRI induced by Eu‐VSOP in vivo [20] were correlated with Prussian blue staining of iron deposition, LA‐ICP‐ToF‐MS to assess Eu‐VSOP deposition in tissue sections [21], histopathology, and proteomics. The study design is shown in Figure 1.

**FIGURE 1:**
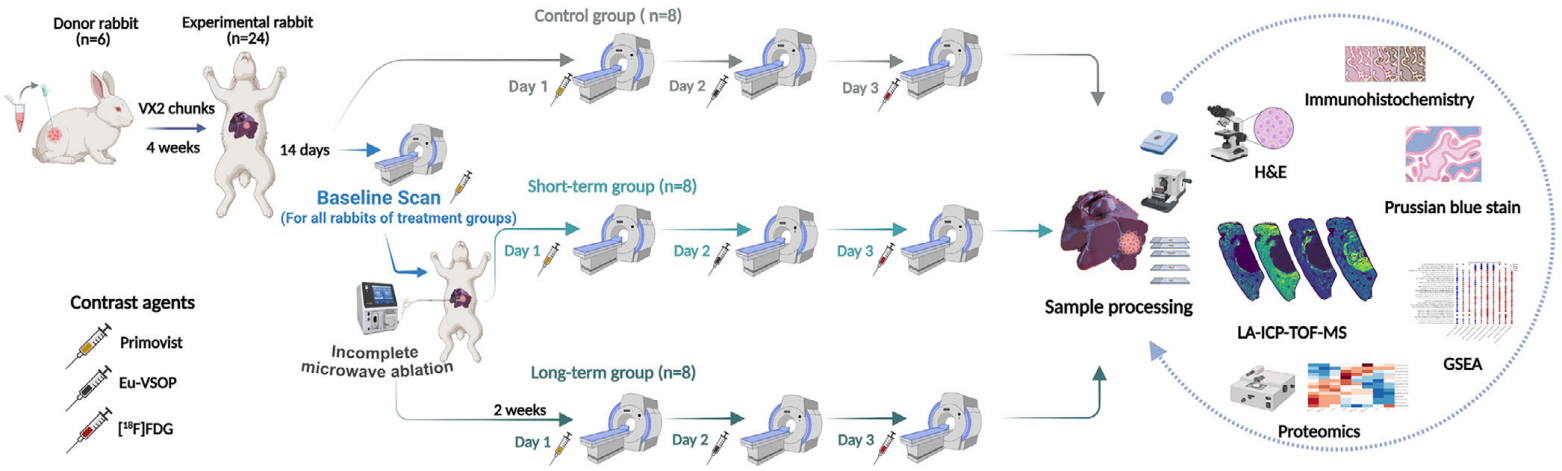
Schematic overview of the translational study design. Thirty New Zealand white rabbits were used, including six donors and 24 VX2 liver tumor‐bearing rabbits randomized into three groups (*n* = 8/group). (1) The control group received no treatment and underwent multiparametric (mp) PET/MRI after 2–3 weeks of tumor growth. (2) The short‐term and (3) the long‐term groups underwent incomplete microwave ablation (iMWA) and received mpPET/MRI 1–3 days (short‐term) and 14–17 days post‐iMWA (long‐term group), respectively. Baseline MRI with Primovist (a hepatocyte‐specific gadolinium‐based contrast agent) was performed in both treatment groups to confirm tumor viability. Experimental imaging was performed over three days: Day 1 – Primovist‐enhanced mpMRI to assess vascularization and viability; Day 2 – intravenous injection of europium‐doped very small superparamagnetic iron oxide nanoparticles (Eu‐VSOP); Day 3 – T2‐weighted MRI 24 h post‐Eu‐VSOP administration to assess peritumoral signal changes, together with [^18^F]FDG‐PET for assessment of tumor glucose metabolism. Following imaging, tissue was collected for ex vivo analyses, including hematoxylin and eosin (H&E) staining, Prussian blue iron staining, laser ablation–inductively coupled plasma time‐of‐flight mass spectrometry (LA‐ICP‐ToF‐MS), and proteomics with gene set enrichment analysis (GSEA).

### 2.2 VX2 Rabbit Liver Tumor Model

This animal study was approved by the Berlin State Office for Health and Social Affairs (LAGeSo) under registration number G150/20. The study was conducted in strict accordance with the regulations of the Federation of European Laboratory Animal Science Associations (FELASA). All the male New Zealand White rabbits (2.5–4.2 kg) supplied by Charles River Laboratories were housed in a laminar‐flow chamber maintained at a constant temperature and humidity, with ad libitum access to feed and water. The VX2 liver tumor model was established as previously described [22, 23]. Following the intrahepatic transplantation of VX2 tumors, standardized clinical welfare assessments were conducted on the rabbits during the 14‐day period of tumor growth and post‐treatment. Animals were evaluated at least once daily for general appearance and behavior, including but not limited to responsiveness, food and water intake, spontaneous activity, posture, and signs of pain, hydration status, urination and defecation, respiratory status, surgical wound condition, and hind‐limb function. If any abnormality was detected, the monitoring frequency was increased according to the severity‐based animal welfare score sheet, generally to two or three assessments per day, and veterinary evaluation and supportive treatment were initiated when indicated. The body weight was assessed relative to each animal's pre‐implantation baseline. A body weight loss of >10% from baseline was considered a welfare‐warning criterion, prompting intensified monitoring and clinical assessment. A body‐weight loss of >20% from baseline or the maximum orthotopic liver tumor diameter of >3 cm or the presence of palpable extrahepatic metastases constituted humane endpoints and required termination of the experiment. Details about tumor implantation and iMWA are described in Appendix S1.

### 2.3 Multiparametric Imaging Protocol

Imaging was conducted using a clinical 3 T PET/MR scanner (Biograph mMR, Siemens) with a knee coil. Mp MRI at all time points included localizer sequences, diffusion‐weighted imaging (DWI) with apparent diffusion coefficient (ADC) mapping, multiplanar T1‐and T2‐weighted imaging and contrast‐enhanced sequences (Appendix S1, Tables S1 and S2).

*Baseline imaging (treatment groups only)* – baseline imaging one day prior to iMWA included T1‐weighted DCE‐MRI after intravenous gadolinium‐based contrast injection (Primovist, 0.1 mmol/kg, Bayer, Germany) to confirm tumor growth presence and viability.

*Experimental imaging (all groups) –* Day 1: mpMRI including DCE with Primovist to assess tumor vascularization and viability; Day 2: mpMRI before and after intravenous administration of Eu‐VSOP injection (0.05 mmol/kg, Berlin, Germany; particle size ∼7‐11 nm [24], Table S3); Day 3: mpPET/MRI, specifically T2‐weighted imaging 24 h post‐Eu‐VSOP, combined with [^18^F]FDG‐PET (FCON, Germany; 80 MBq/rabbit, dynamic acquisition for 10 min and static acquisition at 60 min after injection) to assess tumor viability and metabolic activity.

### 2.4 Quantitative Image Analysis

*Tumor segmentation and mean signal intensities‐* Image analysis was performed using MITK (v2023.12, DKFZ, Germany) and Rover (v3.0.60h, ABX GmbH, Germany) by two radiologists (with > 7 years of experience) in consensus. Several regions of interest (ROIs) were segmented across at least three layers of images to ensure accurate signal extraction excluding vessels and bile ducts. To ensure reproducibility and reduce observer bias in ROI placement, two radiologists independently drew ROIs while blinded to group allocation and pathological results. Intraclass correlation coefficients (ICCs) were computed using a two‐way random‐effects analysis of variance (ANOVA) framework (Table S4). ROIs were defined as follows: *whole tumor* (entire tumor including viable margin and necrotic core (control group) or ablated and non‐ablated tumor post‐iMWA (short‐/long‐term group)), *tumor margin* (viable tumor excluding the necrotic core (control group)), *ablated tumor* (tumor covered by ablation zone post‐iMWA (short‐/long‐term group)), *non‐ablated tumor* (viable residual tumor tissue outside the ablation zone post‐iMWA (short‐/long‐term group)), and *peritumoral rim*. To minimize operator bias, the “peritumoral rim” was defined as the region of low signal intensity on T2‐weighted MRI 24 h after Eu‐VSOP administration, extending 1–3 mm outward from the tumor border. If the rim thickness or signal ratio differed by more than 10%, both readers jointly reviewed and confirmed the ROIs. If consensus could not be reached, a senior radiologist (>10 years of experience) made the final determination. To minimize inter‐individual variability, signal intensities were quantified as ratios using the liver of the same animal as reference. Tumor volume was measured using 3D Slicer by manually delineating the whole‐tumor boundaries on consecutive axial T2‐weighted MR images to generate a three‐dimensional tumor segmentation. Tumor volume was automatically calculated using the Segment Statistics module based on the resulting binary labelmap and expressed in cm^3^. For each rabbit, measurements were performed on the baseline MRI acquired before iMWA and on the corresponding post‐iMWA MRI obtained at the predefined endpoint for the short‐term or long‐term group.

*Functional characterization‐* Tumor vascularization was assessed using DCE‐MRI by comparing ROI values from the arterial and hepatobiliary phases after injection of gadolinium‐based contrast. Tumor viability was evaluated by comparing ADC values before and after treatment using ImageJ. Signal alterations on T2‐TSE MR images induced by Eu‐VSOP accumulation were quantified by measuring the peritumoral signal intensity and rim thickness before (Day 2) and 24 h after Eu‐VSOP administration (Day 3).

Tumor metabolism was quantified by calculating standardized uptake value (SUV) from PET data using the same ROIs applied in Eu‐VSOP analysis, with additional aortic ROIs. The SUV was standardized using the formula: SUV = (ROI activity × body weight)/injected activity.

### 2.5 Tissue Processing and Molecular Analysis

After the imaging endpoint, animals were euthanized, and samples from the tumor, adjacent liver, and contralateral liver were harvested, formalin‐fixed, paraffin‐embedded, digitally scanned, and blindly evaluated. H&E and immunohistochemistry staining were performed. CD206, HLA‐DR, CD11b, CD45, and MMP‐9 were jointly evaluated to characterize immune, myeloid, and extracellular matrix remodeling within the tumor microenvironment. Prussian blue was used to visualize iron deposition from Eu‐VSOP. LA‐ICP‐ToF‐MS analysis enabled elemental mapping of ^153^Eu [21] (Table S5). Proteomics data from liquid chromatography‐tandem mass spectrometry (LC‐MS/MS) were analyzed using a liver metabolic model to evaluate alterations in glucose, lipid, and amino‐acid pathways [25], as well as temporal changes in proteins associated with macrophage activation, myeloid remodeling, and the landscape of immune‐suppressive signature. Details are provided in Appendix S1.

### 2.6 Statistical Analysis

Statistical analyses were performed using GraphPad Prism 10.4.1 (GraphPad Software, Boston, MA, USA) and 4.6.1 (R Foundation for Statistical Computing, Vienna, Austria). Data distribution was assessed with the Shapiro–Wilk test. Results are presented as mean ± SD or median (interquartile range), as indicated in the corresponding figure legends. Two‐group comparisons were performed using two‐tailed paired or unpaired Student's t tests, depending on the study design. For comparisons involving more than two groups, one‐way or two‐way ANOVA followed by Tukey's multiple‐comparisons test was used. When equal variance could not be assumed, Welch's ANOVA with Games–Howell post hoc testing was applied. Statistical procedures specific to HEPATOKIN1‐derived metabolic function analyses are described in the supporting information (Appendix S1, Section 4.4.3). PCA was used for visualization of metabolic profiles. Differences in the overall metabolic profiles were assessed by PERMANOVA with 9,999 permutations, with Benjamini–Hochberg adjustment where appropriate. GO‐BP enrichment was considered significant at FDR < 0.05. Sample sizes are reported in the relevant legends. All tests were two‐sided, with P < 0.05 considered statistically significant.

## 3 Results

### 3.1 Multiparametric [^18^F]FDG‐PET/MRI Confirms Tumor Growth and Residual Tumor Viability After Incomplete Microwave Ablation

Two weeks after intrahepatic tumor implantation, PET/MRI confirmed tumor viability, indicating successful establishment of the model. Specifically, untreated tumors from the control group or pre‐iMWA showed heterogeneous arterial hyperenhancement, delayed hypointensity in the hepatobiliary phase, and diffusion restriction with a small necrotic core (Figure 2A).

**FIGURE 2:**
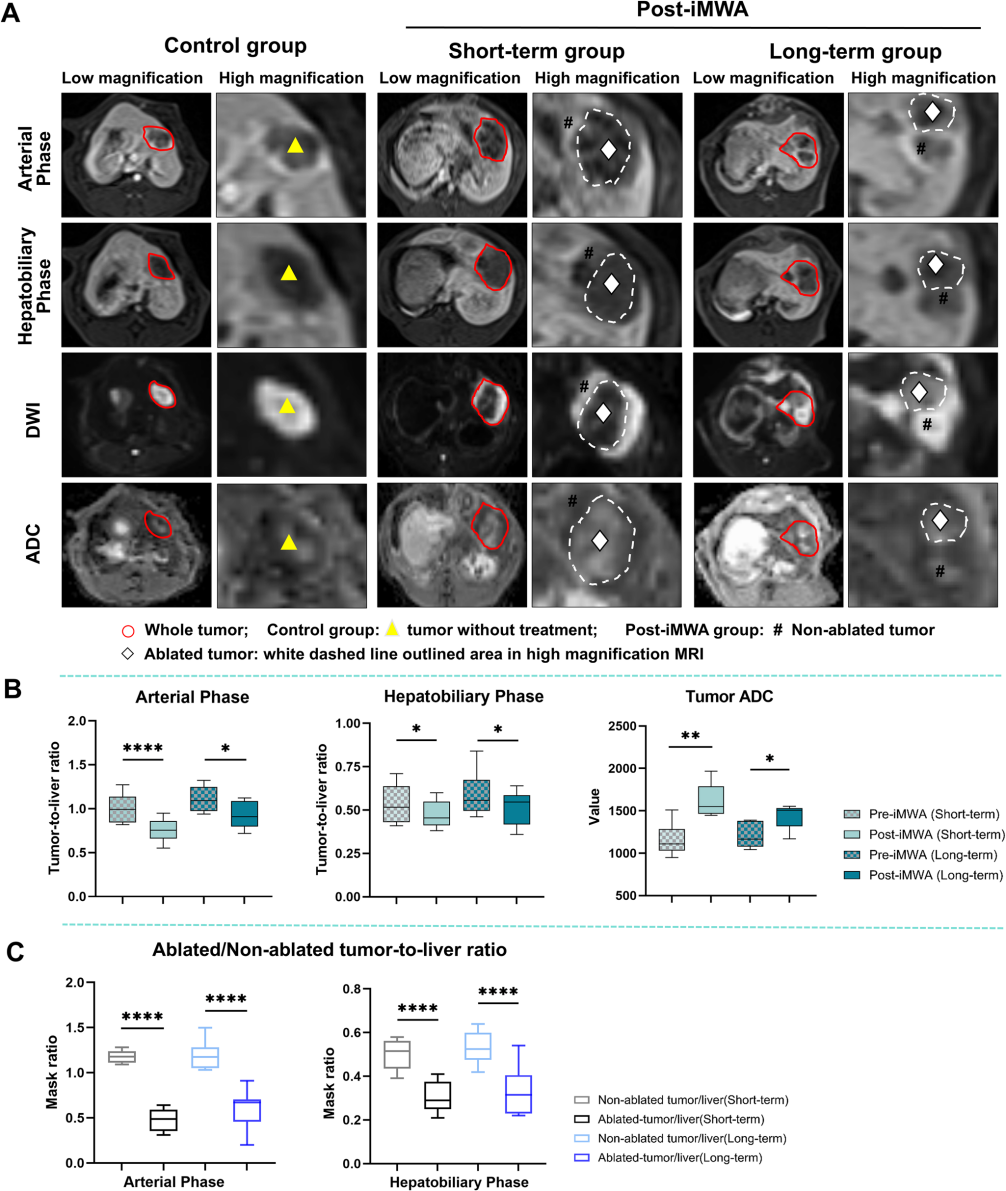
Multiparametric MRI confirms residual tumor viability and vascularization after incomplete microwave ablation (iMWA). (A) Representative MR images from the control group and pre‐/post‐iMWA MR images from the treatment groups, including T1‐weighted arterial‐ and hepatobiliary‐phase images after gadolinium injection, diffusion‐weighted imaging (DWI) with apparent diffusion coefficient (ADC) maps. Control tumors (yellow ▲) remained untreated; in iMWA‐treated animals, non‐ablated residual tumor regions (#) and ablated tumor regions (◇) are indicated and whole tumors are delineated (red contours). Magnified images (right panels) highlight ablated tumor regions (white dashed lines). (B) Quantitative analyses show whole‐tumor‐to‐liver signal intensity ratios on gadolinium‐enhanced images, together with ADC values. (C) Quantitative analyses show ablated vs. non‐ablated tumor‐to‐liver signal intensity ratios on gadolinium‐enhanced images. Data are presented as box‐and‐whisker plots, with the center line indicating the median, the box representing the interquartile range, and the whiskers indicating the minimum and maximum values. *n* = 8 per group. Statistical significance was assessed using two‐tailed paired *t*‐tests. *p < 0.05, **p < 0.01, ****p < 0.0001.

Post‐iMWA, whole tumors in the short‐ and long‐term groups showed significantly reduced arterial enhancement and slightly increased hypointensity in hepatobiliary phase images compared to their baseline scans (Pre‐iMWA) (Figure 2B, arterial phase, short‐term group: *p* < 0.0001; Long‐term group: *p* = 0.0382. Hepatobiliary phase, short‐term group: *p* = 0.0466; Long‐term group: *p* = 0.0130). ADC values were high in the ablated tumor around the needle tract but remained low in the non‐ablated tumor indicating residual viability (pre‐iMWA vs. post‐iMWA; short‐term group *p* = 0.0025; long‐term group, *p* = 0.0364). Accordingly, non‐ablated zones of the tumors post‐iMWA showed significantly more arterial enhancement and contrast uptake in the hepatobiliary phase than the ablated tumor in both the short‐term group (Figure 2C, non‐ablated tumor vs. ablated tumor‐to‐liver ratio in arterial phase: *p* < 0.0001; hepatobiliary phase: *p* < 0.0001) and long‐term group (arterial phase: *p* < 0.0001; hepatobiliary phase: *p* < 0.0001). Meanwhile, transient body‐weight fluctuations were observed in some rabbits following tumor implantation or iMWA. However, longitudinal monitoring showed no marked weight loss, with body weight remaining generally stable and gradually increasing over the study period (Figure S1).

[^18^F]FDG PET/MRI (Figure 3A) further demonstrated persistent metabolic activity in residual tumor following iMWA. SUVmean of static PET showed a reduced uptake in ablated compared to non‐ablated tumors both in the short‐term and long‐term groups. Tumor SUVmean (Figure 3B) in the control group was 2.305 ± 0.937 and SUVmean of the whole tumor (including treated and untreated zone) in short‐term and long‐term groups were 1.267 ± 0.640, and 1.163 ± 0.396, respectively. In the short‐term group, reduced central [^18^F]FDG uptake around the needle track was consistent with effective partial ablation, while persistent [^18^F]FDG uptake in untreated zones reflected remaining viable tumor (SUVmean of non‐ablated tumor = 1.915 ± 0.841, ablated tumor = 0.374 ± 0.134). In the long‐term group, [^18^F]FDG uptake remained higher in non‐ablated tumor than in ablated tumor regions (SUVmean of non‐ablated tumor = 1.602 ± 0.910, ablated tumor = 0.591 ± 0.126). Meanwhile, MRI‐based volumetry demonstrated heterogeneous longitudinal changes in lesion volume after iMWA. Relative volume changes were generally modest or variable at the early post‐iMWA time point (short‐term group), whereas larger increases were observed in several animals at the later time point (long‐term group) (Figure S2). The [^1^ ^8^F]FDG SUVmean ratio between ablated tumor and non‐ablated tumor areas of the two treatment groups was also analyzed (Figure 3C). It revealed that the long‐term group showed partial metabolic recovery, with a higher SUVmean ratio than the short‐term group (*p* <0.05). Meanwhile, the early dynamic SUV curves (Figure S3) revealed a time‐dependent redistribution of [^18^F]FDG uptake, with tracer activity progressively shifting from liver parenchyma toward the tumor tissue, particularly in the residual viable tumor zones. This redistribution pattern was most pronounced in the long‐term group at two weeks post‐ablation. These findings suggest that a relative increase in metabolic recovery occurs in non‐ablated tumor regions over time, potentially reflecting contributions from immune cell infiltration or residual tumor activity.

**FIGURE 3:**
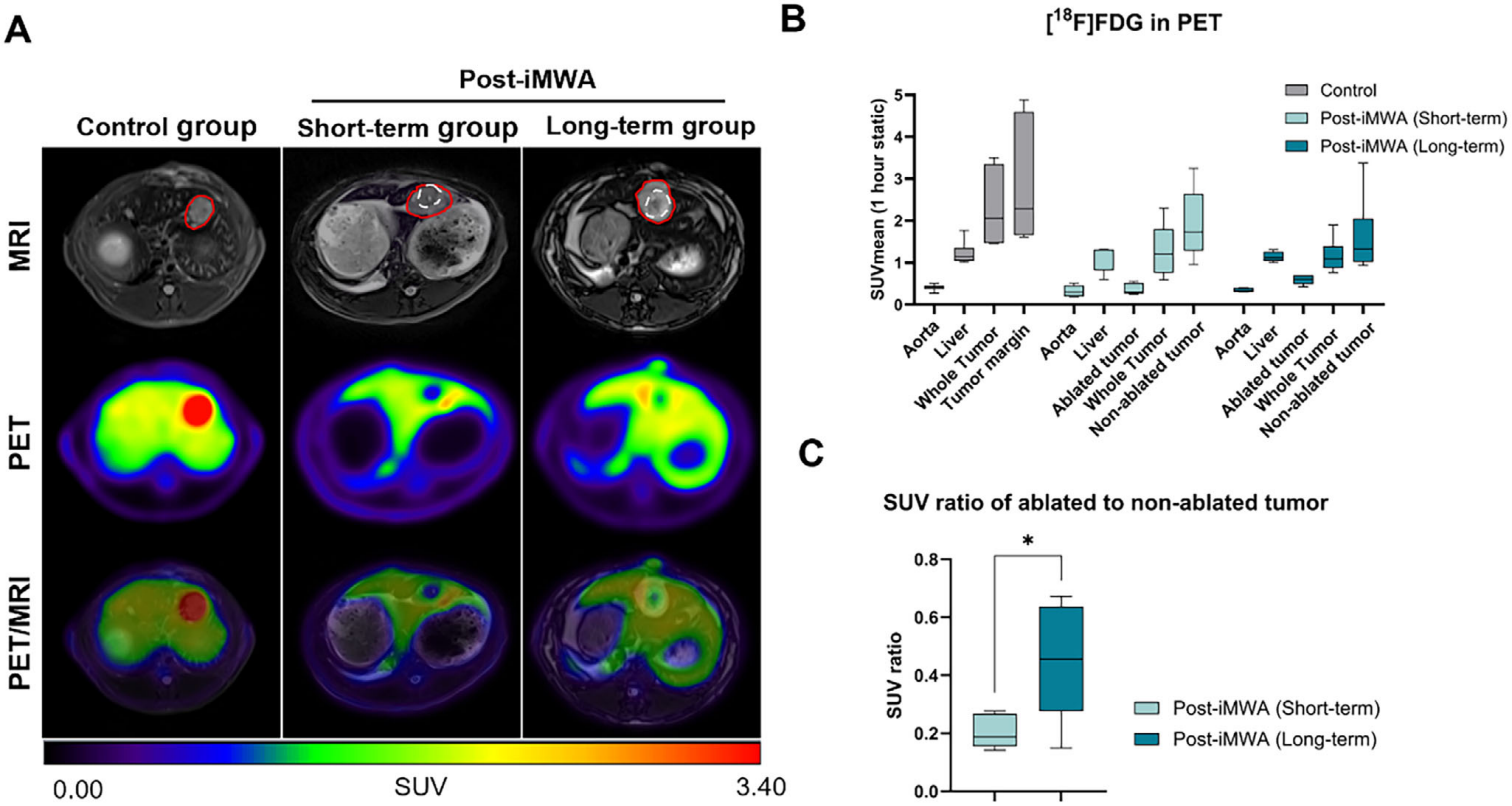
[^18^F]FDG‐PET/MRI demonstrates residual tumor viability and glycolytic activity after incomplete microwave ablation (iMWA). (A) Representative [^18^F]FDG‐PET/MRI images from the control, short‐term, and long‐term groups. On MR images, red contours delineate the whole tumor, whereas white dashed contours indicate the ablated region in the treatment groups. The region between the two contours represents non‐ablated viable residual tumor. [^18^F]FDG‐PET reflects glucose uptake expressed as standardized uptake value (SUV) (red = higher uptake, blue = lower uptake). (B) Quantification of [^18^F]FDG SUVmean across tissue compartments and experimental groups, including the aorta, liver, whole tumor, viable tumor margin (excluding central necrosis in control tumors), ablated tumor regions, and non‐ablated tumor regions (control: *n* = 6, short‐term: *n* = 5, long‐term: *n* = 6). (C) Box‐and‐whisker plots illustrate the SUVmean ratio between ablated and non‐ablated regions in the short‐term and long‐term groups. The ablated‐to‐non‐ablated SUVmean ratio was significantly higher in the long‐term group than in the short‐term group (*p* = 0.0267; short‐term: *n* = 5, long‐term: *n* = 6). Statistical significance was assessed using a two‐tailed unpaired *t*‐test. Data in (B) and (C) are presented as box‐and‐whisker plots, with the center line indicating the median, the box representing the interquartile range, and the whiskers indicating the minimum and maximum values.

### 3.2 iMWA Induces a Transition Toward a Macrophages‐Enriched, CD206‐Associated Tumor Environment

Histopathological and immunohistochemical analyses were subsequently performed to confirm tumor survival following iMWA and to characterize the immune profiling associated with surrounding tissue remodeling (Figure 4A). H&E staining confirmed the partial tumor survival and regrowth in treatment groups, which was consistent with the imaging evidence shown above of residual viable tumor following iMWA.

**FIGURE 4:**
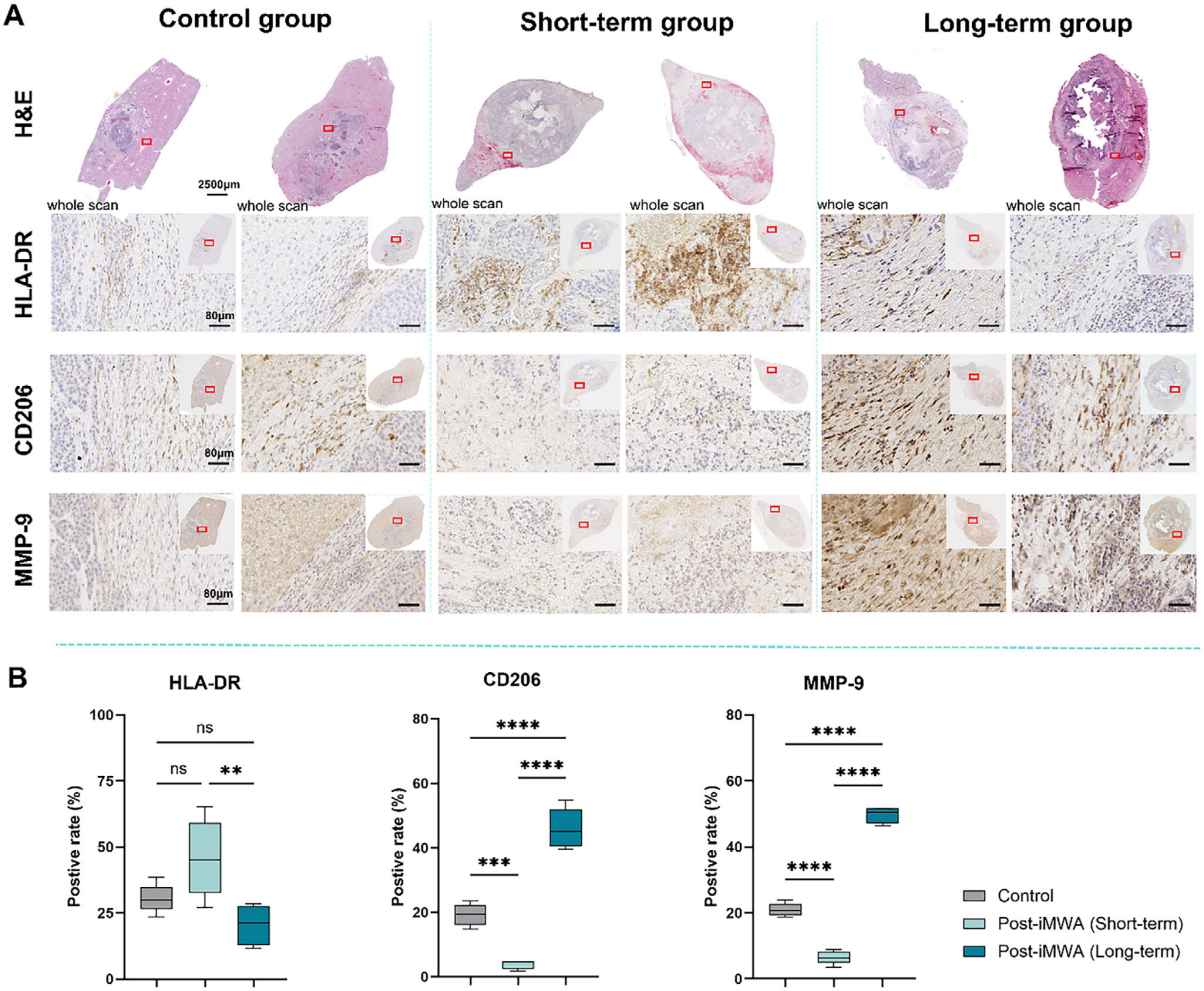
Immunohistochemistry reveals immunosuppressive remodeling of the tumor microenvironment following incomplete microwave ablation (iMWA). (A) Representative whole‐slide H&E staining and immunohistochemistry for HLA‐DR, CD206, and MMP‐9 across study groups. (B) Quantification of HLA‐DR, CD206, and MMP‐9 expressed as the percentage of positively stained area. Data are presented as box‐and‐whisker plots, with the center line indicating the median, the box representing the interquartile range, and the whiskers indicating the minimum and maximum values (*n* = 5 per group). Statistical significance was assessed using one‐way ANOVA followed by Tukey's multiple‐comparisons test. **p < 0.01; ***p < 0.001; ****p < 0.0001; ns, not significant.

Immunohistochemical analysis revealed immunological remodeling and metastatic tendency post‐iMWA (Figure 4B). Specifically, the expression of CD206 and MMP‐9, which are associated with immunosuppression, extracellular matrix degradation, and tissue‐invasive remodeling, was significantly decreased in the short‐term group compared with the control group (CD206, *p* = 0.0002; MMP‐9, *p* < 0.0001) but was significantly elevated in the long‐term group (CD206, *p* < 0.0001; MMP‐9, *p* < 0.0001) compared to controls. HLA‐DR, CD11b, and CD45 were further evaluated to characterize antigen‐presentation status, myeloid‐related changes, and overall leukocyte infiltration, respectively. HLA‐DR expression showed a trend toward higher levels in the short‐term group compared to control (*p* = 0.0743) but decreased again in the long‐term group (short‐term vs. long‐term: *p* = 0.0043). CD11b and CD45 were downregulated shortly after iMWA and increased again in the longer term (Figure S4, Short‐term vs. Long‐term: CD11b: *p* < 0.0001; CD45: *p* < 0.0001). Overall, these findings suggest that iMWA first induces early inflammatory dysregulation, which subsequently triggers late‐stage pathology characterized by the development of a macrophage‐rich, CD206‐associated immunosuppressive microenvironment with MMP‐9‐associated matrix remodeling and broader reorganization of myeloid and leukocyte populations.

### 3.3 Preferential Eu‐VSOP Accumulation in the Macrophage‐Rich, CD206‐Associated Peritumoral Rim

Given the progressive increase of CD206 expression observed longitudinally over time following iMWA, along with the remodeling of the immunosuppressive microenvironment. Based on our previous in vitro studies [16] and the pathological and immunological changes observed in the in vivo studies described above, we next evaluated whether Eu‐VSOP‐enhanced MRI could noninvasively visualize macrophage‐associated peritumoral remodeling and immunosuppressive microenvironment in vivo.

T2‐weighted MRI 24 h after Eu‐VSOP injection (Day 3) revealed a hypointense peritumoral rim in all groups (Figure 5A). In the control and short‐term groups, the mean rim‐to‐liver signal ratio decreased from >1 pre‐injection to <1 post‐injection (Figure 5C, control: *p* = 0.0011; short‐term: *p* = 0.0288). This effect was more pronounced in the long‐term group (*p* < 0.0001), indicating preferential Eu‐VSOP accumulation within the peritumoral rim. Additionally, the hypointense rim was significantly thicker (Figure 5D) in the long‐term group compared to both the control (*p* = 0.0015) and short‐term group (*p* = 0.0019), while no difference was observed between the control and short‐term groups (*p* > 0.99).

**FIGURE 5:**
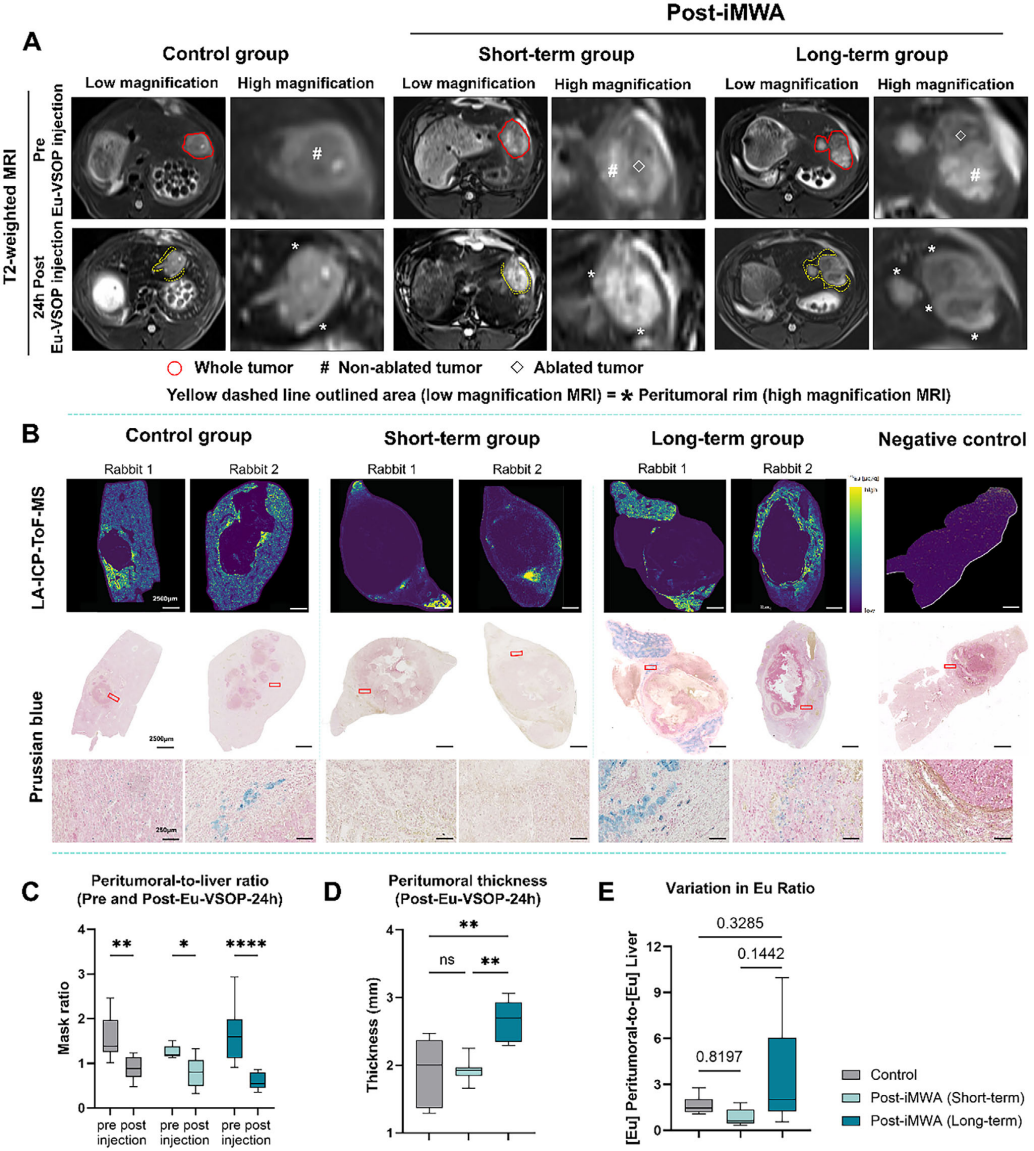
Eu‐VSOP‐enhanced molecular MRI and ex vivo validation reveal peritumoral nanoparticle enrichment. (A) Representative axial T2‐weighted MR images acquired before and 24 h after Eu‐VSOP injection. Hypointense peritumoral rims (*) were observed after Eu‐VSOP injection in all groups. Red contours delineate the whole tumor; post‐iMWA, (#) indicates non‐ablated tumor regions and (◇) indicates ablated tumor regions. Yellow dashed outlines on low‐magnification images indicate the peritumoral rim corresponding to the regions marked by asterisks (*) on the high‐magnification images, which highlight the morphology of the rim and its more pronounced appearance in the long‐term group. (B) Ex vivo validation of Eu‐VSOP deposition. Elemental mapping using LA‐ICP‐ToF‐MS shows the spatial distribution of ^153^Eu (top row), while Prussian blue staining demonstrates iron deposition in the corresponding peritumoral regions (middle and bottom rows), showing spatial correspondence with the hypointense peritumoral rim on MRI. For each experimental group, two independent biological replicates from two different rabbits are shown. Each column represents one rabbit with the corresponding ^153^Eu mapping and iron deposition displayed in parallel. The rightmost column shows a negative‐control rabbit that did not receive Eu‐VSOP. (C) Quantification of peritumoral rim‐to‐liver signal intensity ratios pre‐ and 24 h post‐Eu‐VSOP (control: *n* = 8, short‐term: *n* = 8, long‐term: *n* = 7; Statistical significance was performed by two‐way ANOVA with Tukey's multiple‐comparisons test, *p < 0.05; **p < 0.01; ****p < 0.0001; ns, not significant). (D) Quantification of peritumoral rim thickness 24 h after Eu‐VSOP administration (control: *n* = 8, short‐term: *n* = 8, long‐term: *n* = 7; Statistical significance was performed by one‐way ANOVA with Tukey's multiple‐comparisons test, **p <.01). (E) Quantified ^153^Eu peritumoral‐to‐liver ratios across groups. Data are presented as box‐and‐whisker plots, with the center line indicating the median, the box representing the interquartile range, and the whiskers indicating the minimum and maximum values. (control: *n* = 6, short‐term: *n* = 5, long‐term: *n* = 6; Statistical significance was performed by one‐way ANOVA with Tukey's multiple‐comparisons test).

Eu‐VSOP localization was validated ex vivo by LA‐ICP‐ToF‐MS and Prussian blue staining (Figure 5B), which further demonstrated the preferential deposition of Eu‐VSOP in the macrophage‐rich peritumoral zone on a molecular level. Specifically, LA‐ICP‐ToF‐MS revealed spatial enrichment of ^1^ ^5^ ^3^Eu in the peritumoral region, consistent with the hypointense rim observed on T2‐weighted MRI. Prussian blue staining further confirmed iron deposition in the corresponding regions. More importantly, the previous immunohistochemical analysis showed that the regions of increased CD206 expression spatially corresponded with areas of Eu and iron enrichment detected by LA‐ICP‐ToF‐MS and Prussian blue staining. Furthermore, the trend in the CD206+ rate was consistent with the ROI signal of Eu‐VSOP‐enhanced MRI, thickness (Figure 5D), and variation in Eu ratio (Figure 5E). Quantitatively, the ^153^Eu peritumoral‐to‐liver ratio was markedly higher in the long‐term (3.459 ± 3.483) than in the short‐term group (0.848 ± 0.576), confirming increased Eu‐VSOP accumulation in the peritumoral rim over time. As a negative control, one rabbit from the long‐term group did not receive Eu‐VSOP and showed no detectable ^153^Eu or iron signal on LA‐ICP‐ToF‐MS or Prussian blue staining (Figure 5B). In summary, these findings support the role of Eu‐VSOP‐enhanced MRI as a spatial imaging biomarker for macrophage‐rich, CD206‐associated peritumoral remodeling following iMWA.

### 3.4 Proteomic Profiling Supports Immunometabolic Reprogramming After iMWA

Proteomics analysis (Figure 6) revealed distinct metabolic profiles among viable tumor tissues, ablated tumor regions, and healthy liver tissues. Specifically, unsupervised hierarchical clustering (Figure 6A) revealed substantial heterogeneity among liver, untreated tumor, ablated tumor, and viable non‐ablated residual tumor tissues; distinct patterns were observed across oxidative, carbohydrate, amino acid, lipid, and ketone body metabolic functions. Principal component analysis (PCA) of oxygen uptake, lactate exchange, glucose exchange, and glutamate uptake was subsequently performed on residual tumors from the three groups (Figure 6B). PC1 and PC2 accounted for 52.0% and 32.6% of the total variance, respectively, together explaining 84.6% of metabolic variability. Residual tumors in both post‐iMWA groups showed modest displacement relative to untreated control tumors, although substantial overlap remained. Gene ontology biological process (GO‐BP) analysis further demonstrated spatial and temporal heterogeneity in the immune‐inflammatory response after iMWA (Figure 6C). In the short‐term group post‐ablation, IL‐6 production in ablated tumor tissue showed significant positive enrichment (ES = 0.539, *P* = 0.0012, FDR = 0.042). In contrast, both “immune effector process” and “inflammatory response” showed significant negative enrichment in the ablated tissue of the long‐term group. In addition, among the individual metabolic functions in the model, oxygen uptake showed the clearest temporal pattern. Oxygen uptake (Figure 6D) in tumors within the ablation zone gradually decreased over time, and viable non‐ablated residual tumor also exhibited a similar trend. Lactate exchange (Figure 6E) likewise showed a trend following iMWA, but with greater interindividual variability. These findings suggest that substrate utilization and processing undergo heterogeneous metabolic remodeling following iMWA. A STRING‐based protein–protein interaction (PPI) network (Figure 6F) revealed extensive links between the macrophage regulatory program and TGF‐β signaling, inhibitory immune pathways, antigen presentation, oncogenic signaling, lectin‐related biological functions, and metabolic regulation. This suggests coordinated interactions between immune and metabolic processes in the post‐ablation microenvironment. The Stouffer heatmap (Figure 6G) provided further insight into these dynamics. On Day 3, the M1‐like macrophage activation pathway showed a positive score, whereas immune checkpoint tolerance and adenosine‐mediated immunosuppression showed distinctly negative scores. By Day 14, this pattern had shifted substantially: M1‐like macrophage activation was markedly suppressed, while immune checkpoint tolerance shifted to a distinctly positive state, and adenosine‐mediated immunosuppression also shifted from negative to positive. At the same time, TCR‐associated T‐cell activation remained suppressed. Overall macrophage activation and monocyte‐to‐macrophage differentiation were enhanced by Day 14. Together with imaging and histological findings, the proteomic profile reveals that the Eu‐VSOP‐associated peritumoral microenvironment reflects a broader post‐ablation remodeling program involving macrophage ‐associated immune reorganization, matrix remodeling, and metabolic adaptation.

**FIGURE 6:**
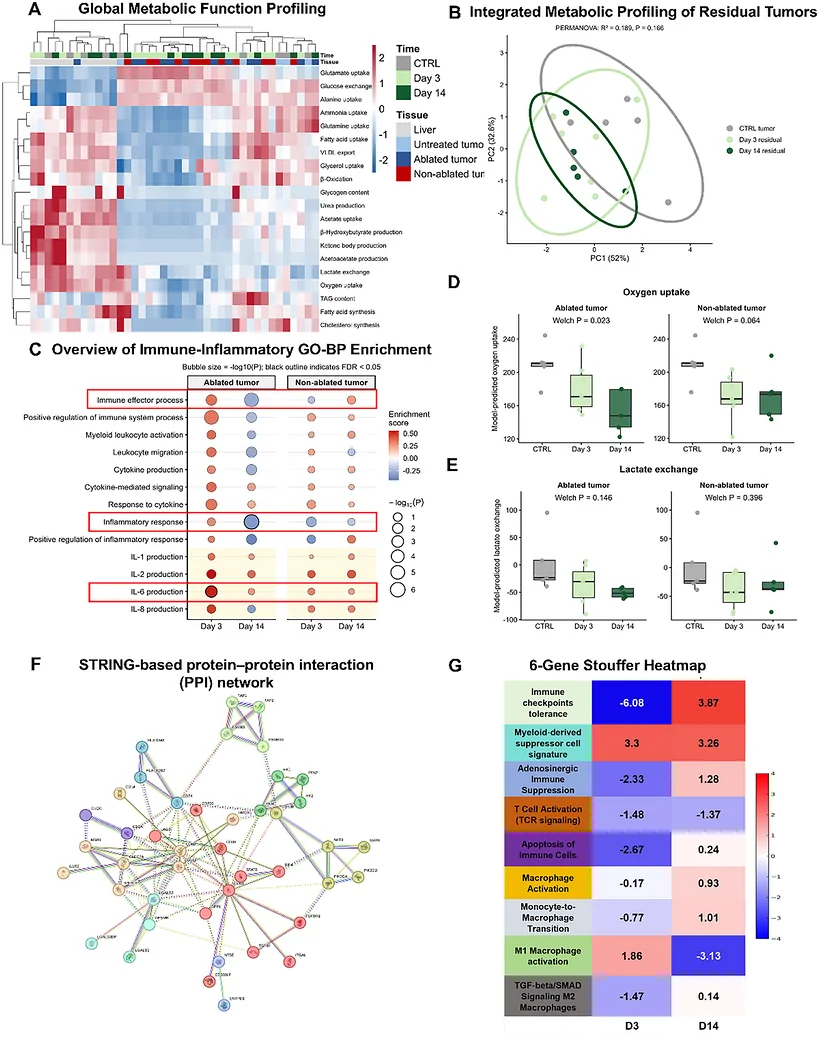
Proteomics reveals immunometabolic reprogramming and macrophage activation following incomplete microwave ablation (iMWA). (A) Global metabolic function profiling based on proteomics‐derived, sample‐specific metabolic modeling. Metabolic functions were standardized to row‐wise Z‐scores and hierarchically clustered using Euclidean distance and average linkage. Z‐scores were truncated at −2.5 and +2.5 for visualization. Samples (*n* = 40) comprised the control liver and tumor, as well as liver, ablated tumor, and non‐ablated tumor on Day 3 and Day 14. Annotation bars indicate experimental time point and tissue compartment. (B) Principal component analysis (PCA) of the integrated metabolic phenotype of viable residual tumors based on oxygen uptake, lactate exchange, glucose exchange, and glutamate uptake. Differences in overall metabolic profiles were assessed by PERMANOVA, with Benjamini–Hochberg correction where appropriate; homogeneity of multivariate dispersion was additionally assessed. (C) GO Biological Process (GO‐BP) enrichment analysis of immune‐ and inflammation‐related pathways in ablated and non‐ablated tumor compartments at Day 3 and Day 14 relative to untreated control tumor. Bubble color represents the enrichment score (ES; red, positive ES; blue, negative ES), and bubble size represents −log_10_(P). Black outlines indicate pathways with FDR < 0.05. (D) Model‐predicted oxygen uptake in ablated and non‐ablated tumor compartments. Individual points represent individual tissue samples. Boxplots indicate the median and interquartile range, with whiskers extending to 1.5× the interquartile range. (E) Model‐predicted lactate exchange in ablated and non‐ablated tumor compartments. Group definitions were identical to those in (D). (F) STRING‐based protein–protein interaction (PPI) network of 46 curated proteins associated with macrophage biology and tumor immune regulation. Full protein and cluster assignments are provided in Table S7. (G) Six‐gene Stouffer Z‐scores heatmap. Composite Z‐scores were calculated from predefined sixgene signature sets using proteomic data at Day 3 (D3) and Day 14 (D14) post‐iMWA. Positive Z‐scores indicate relative activation, and negative Z‐scores indicate relative suppression compared with baseline conditions. Signature definitions are provided in Table S6. CTRL tumor, whole tumor in control group; D3 (or D14), Day 3 (or Day 14) post‐iMWA; ES, enrichment score; FDR, false discovery rate.

## 4 Discussion

This translational study demonstrates that incomplete ablation of liver tumors induces a temporally evolving, pro‐tumorigenic TME characterized by coordinated immune, myeloid, and metabolic remodeling. This immunosuppressive remodeling was visualized noninvasively by Eu‐VSOP‐enhanced MRI. Compared with elevated HLA‐DR and lower CD206 expression shortly after iMWA, growing residual tumors at two weeks exhibited increased CD206 and MMP‐9 expression and reduced HLA‐DR levels, consistent with a shift from an early inflammatory response toward an immunosuppressive, tumor‐promoting phenotype. These findings suggest that incomplete ablation may promote tumor progression not only through the persistence of viable tumor cells but also through active remodeling of the surrounding TME. Importantly, Eu‐VSOP‐enhanced MRI enabled the in vivo detection of this remodeling, with preferential nanoparticle accumulation in macrophage‐rich, CD206‐associated peritumoral regions at two weeks, highlighting its potential as a noninvasive biomarker of TME remodeling following incomplete ablation.

These results fit within a broader body of evidence linking sublethal heat exposure during ablation to immune evasion. Prior studies showed that incomplete ablation can activate oncogenic and immunosuppressive signaling (e.g., PI3K/mTOR, O‐GlcNAcylation) and suppress T‐cell activity [9, 10], ultimately accelerating tumor growth and resistance to PD‐1 blockade [11]. Our findings extend this understanding by providing in vivo molecular imaging evidence. Specifically, T2‐weighted MRI after Eu‐VSOP injection consistently revealed a hypointense peritumoral rim that was most pronounced two weeks following iMWA. This imaging pattern correlated with ex vivo europium quantification (via LA‐ICP‐ToF‐MS), iron staining (Prussian blue), and CD206 immunohistochemistry [26, 27], supporting preferential Eu‐VSOP accumulation within macrophage‐rich, CD206‐associated peritumoral regions.

Meanwhile, Stouffer‑derived pathway profiles and the PPI interactome provided mechanistic insight into dynamic TME remodeling after iMWA. Concordant with the imaging and histological findings, proteomic scores revealed a phenotypic shift from early M1 biased inflammation to a myeloid‐dominated immunosuppressive state. Markedly diminished M1‐like macrophage activation, coupled with up‑regulated immune checkpoint tolerance and adenosinergic‐suppressive signatures at day 14, demonstrates how residual tumors rewire innate immunity to evade antitumor surveillance. The PPI network highlights coordinated pathway crosstalk: macrophage‑centric modules are closely linked to TGF‑β signaling, immune checkpoint mediators, antigen presentation machinery, and glycolytic metabolism. This indicates that macrophage activation and myeloid reprogramming are embedded within a regulatory circuit integrating myeloid, immune‐inhibitory and metabolic programs. Such molecular interplay may drive residual tumor regrowth, with myeloid cells serving as hubs for diverse pro‑tumorigenic signals.

Given the increasing use of ablation in both primary and secondary liver malignancies, insights from this study are highly clinically relevant. Although non‐thermal or non‐heat‐based ablation modalities may elicit different immune responses within the TME than MWA [28, 29], ablation is generally thought to induce immunogenic cell death and stimulate antitumor responses, whereas incomplete treatment may instead foster immune evasion [11]. Thus, strategies such as CSF1R inhibition [30] could complement ablation in an adjuvant setting to reduce the risk of recurrence. Adjuvant immunotherapy following curative‐intent local treatment, as exemplified by the IMbrave050 trial, showed encouraging results in early analyses [31]. However, an updated follow‐up recently demonstrated that this benefit was not sustained [32]. These findings further suggest that patient selection, treatment timing, and TME characteristics may be critical determinants of therapeutic benefit.

Thus, molecular imaging could help stratify patients based on their TME`s composition or monitor response, suggest suitable immunomodulatory therapies or guide adjuvant treatment timing, particularly in the common clinical scenario of unintended incomplete ablation. This study supports the feasibility of molecular MRI for characterizing a macrophage‐rich peritumoral niche in vivo. Specifically, Eu‐VSOP‐enhanced MRI enabled noninvasive assessment of spatially organized post‐ablation remodeling, with preferential nanoparticle accumulation in macrophage‐rich peritumoral regions which demonstrated immunosuppressive traits. Compared with molecular imaging approaches based on PET tracers or gadolinium‐labeled antibodies targeting immune markers such as CD8 or HLA‐DR [33, 34], Eu‐VSOP may offer complementary translational potential by combining high‐spatial‐resolution MRI with ex vivo elemental validation. Our previous in vitro study [16] and these in vivo findings suggest that M2‐preferential Eu‐VSOP enrichment may be facilitated by increased glycosaminoglycan (GAG) abundance and a thicker macrophage glycocalyx on M2‐ compared to M1‐like macrophages. In line with this, Taupitz et al. [35] proposed that in vivo the citrate coating is functionally replaced by the interactions between the iron oxide surface and GAGs, owing to their higher chelating capacity when compared to citrate. More broadly, iron oxide nanoparticles circulate in the blood and accumulate in phagocytosing cells. Notably, ferumoxytol (FERABRIGHT) has recently received FDA approval as an iron‐based MRI contrast agent for brain imaging in adults with known or suspected malignant brain neoplasms [36]. Moreover, Ferucarbotran (Resovist, Bayer) has also been used clinically for contrast‐enhanced liver MRI [37], providing a clinical precedent for the diagnostic use of iron oxide nanoparticles. Iron oxide nanoparticles are also being investigated for theranostic applications, including the delivery of immunomodulatory payloads (e.g., siRNA targeting PD‐L1) [38]. However, despite this potential, several steps are required before clinical translation. First, although Eu‐VSOP offers dual‐modality imaging with elemental verification, the long‐term biodistribution, clearance, and biocompatibility of Fe/Eu in humans require further toxicological evaluation, and GMP‐compliant production will be necessary. In addition, key operational parameters (i.e., Eu‐VSOP dosing, injection timing, and imaging windows) will require systematic optimization to ensure clinical applicability. Third, implementation of Eu‐VSOP‐enhanced molecular MRI in clinical workflows will require careful validation across scanner platforms, pulse sequences, and existing contrast‐agent protocols, together with standardized methods for reproducible delineation of peritumoral regions in human liver tumors. Finally, extended longitudinal cohorts will be required to determine whether these imaging features can robustly track long‐term post‐ablation microenvironmental remodeling and its relationship to residual tumor progression.

Our findings further support the role of immunometabolic crosstalk in post‐ablation tumor persistence. Tumor tissues demonstrated altered oxidative and glycolytic metabolic functions compared with liver parenchyma, while [^18^F]FDG PET showed persistent metabolic activity within viable residual tumor tissue. Increased reliance on glycolytic metabolism can contribute to an acidic and nutrient‐competitive microenvironment that impairs T‐cell function and favors immunosuppressive myeloid states [39]. In our study, the evolving regional distribution of [^18^F]FDG uptake, together with proteomic evidence of altered oxidative metabolism, cytokine signaling, immune‐checkpoint tolerance, and adenosinergic suppression, illustrates how metabolic and immune programs converge within the residual TME after iMWA. These coordinated changes may contribute to the establishment of an immunosuppressive niche that is permissive to residual tumor regrowth.

Despite these promising results, several limitations must be acknowledged. First, the relatively small sample size may limit the generalizability of the results, particularly for LA‐ICP‐ToF‐MS and proteomic analyses, in which substantial inter‐animal heterogeneity was observed. Larger cohorts incorporating spatially stratified tissue sampling will be required to better characterize this biological variability and validate the observed molecular patterns. Second, although the VX2 rabbit model is well established and widely accepted as a preclinical liver cancer model in interventional radiology, it does not fully reproduce the biological background of human hepatocellular carcinoma, particularly chronic liver disease and cirrhosis. Nevertheless, VX2 tumors reproduce several imaging and glycolytic features relevant to liver malignancies and provide a technically suitable platform for image‐guided ablation studies. However, the metabolic proteomics liver modeling used in this study was originally validated for HCC and was adapted to assume differences between VX2 tumors and liver parenchyma similar to those in HCC. Third, the spatial resolution of molecular imaging could be further improved, e.g., by using optimized receiver coils or quantitative relaxometry approaches to enhance sensitivity to subtle signal changes. Fourth, the follow‐up period was limited to 14–17 days post‐iMWA. Although macrophage‐rich peritumoral remodeling, increased CD206 expression, and iron deposition were already evident at this time point, extended longitudinal studies may be needed to determine whether the observed macrophage‐rich immunosuppressive niche persists, resolves, or further evolves during future residual tumor progression. Finally, although Eu‐VSOP enrichment showed close spatial correspondence with macrophage‐rich, CD206‐associated peritumoral regions, the present study does not establish cell‐type‐specific uptake in vivo. CD206 is not exclusively expressed by macrophages, and uptake by Kupffer cells, other macrophage subsets, dendritic cells, or other phagocytic populations therefore cannot be excluded. Future studies incorporating multiplex immunofluorescence, a broader range of TAM phenotypes, and cell‐resolved spatial analyses will be important for defining the cellular basis of Eu‐VSOP enrichment and its utility as an imaging biomarker.

## 5 Conclusions

In conclusion, this study provides a comprehensive framework for investigating post‐ablation immunometabolic remodeling in vivo. Residual liver tumors after incomplete ablation developed a macrophage‐rich peritumoral microenvironment with increased CD206 expression and other features suggesting immunosuppression. Eu‐VSOP‐enhanced molecular MRI revealed preferential nanoparticle accumulation in these regions, providing a noninvasive imaging correlate of the changes identified by histopathologic and proteomic analyses. Thus, such molecular imaging approaches may complement conventional MRI by identifying microenvironments associated with adverse biological remodeling, thereby guiding risk stratification and informing the timing and selection of adjuvant therapies after incomplete ablation of liver tumors.

## Author Contributions

**Yubei He**: conceptualization, methodology, investigation, writing – original draft, software, validation, visualization, data curation, formal analysis. **Han Xu**: methodology, investigation, validation, software, data curation, formal analysis, project administration. **Julian Rogasch**: software, visualization, methodology. **Nikolaus Berndt**: methodology, data curation, software, formal analysis. **Jing Guo**: methodology, software, formal analysis. **Eyk Schellenberger**: methodology, software. **Akvile Häckel**: investigation, validation, methodology. **Luisa Heidemann**: methodology, investigation, validation. **Friederike Hesse**: investigation, validation, methodology. **Yu Liu**: validation, software, formal analysis, methodology. **Justus Ramtke**: methodology, investigation, validation. **Nicola Beindorff**: methodology, data curation. **Frank Hofheinz**: validation, methodology. **Avan Kader**: methodology, software. **Michael Mülleder**: methodology, software, formal analysis, data curation, investigation. **Kathrin Textoris–Taube**: investigation, validation, methodology. **Bernd Hamm**: project administration, supervision, resources, funding acquisition. **Dominik N. Müller**: writing – review and editing, supervision, resources, project administration. **Robin Schmidt**: methodology. **Ralf Hauptmann**: methodology, software. **Heike Traub**: methodology, software, formal analysis. **Mathias Schannor**: methodology, software, formal analysis, investigation, validation. **Lynn Jeanette Savic**: writing – review and editing, funding acquisition, conceptualization, project administration, resources, supervision, data curation, methodology.

## Funding

This work was funded by the Deutsche Forschungsgesellschaft (DFG, German Research Foundation) through the CRC 1340 “Matrix in vision” (Project‐ID 372486779, SFB 1340/2 and 1340/3 2026; projects B08 and C02) and the research unit FOR5628 “Onco‐MRE” (Project‐ID513752256; projects B02 and A02).

## Ethics Approval and Consent to Participate

This animal study was approved by the Berlin State Office for Health and Social Affairs (LAGeSo) under registration number G150/20. The study was conducted in strict accordance with the regulations of the Federation of European Laboratory Animal Science Associations (FELASA).

## Use of Generative AI and AI‐Assisted Technologies in the Writing Process

OpenAI was used solely for English‐language editing and improvement of readability during manuscript preparation.

## Conflicts of Interest

Julian M. M. Rogasch has received speaker honoraria from Hexal AG, GE Healthcare, and Novartis and has served on an advisory board for Novartis. (None of these activities are related to the research presented in this paper). The other authors declare no conflicts of interest.

## Supporting information




**Supporting File**: advs78490‐sup‐0001‐SuppMat.docx.

## Data Availability

The datasets used and/or analysed during the current study are available from the corresponding author on reasonable request.
