# Supplementary material for: Molecular MRI Reveals Macrophage‐Enriched Peritumoral Immune Remodeling After Incomplete Ablation in Liver Cancer
Source: Adv Sci (Weinh). 2026 Nov 5:e78490. Online ahead of print. doi: 10.1002/advs.78490 (PMC13735433; doi:10.1002/advs.78490)
Supplement: Supplementary file 1 [file ADVS-9999-e78490-s001.docx]

Supporting Information

Title

Molecular MRI Reveals Macrophage‐Enriched Peritumoral Immune Remodeling After Incomplete Ablation in Liver Cancer

Yubei He ^1,2,‡^, Han Xu ^1,‡^, Julian M. M. Rogasch ^3^, Nikolaus Berndt ^1^,^4^ , Jing Guo ^1^, Eyk Schellenberger ^1^, Akvile Häckel ^1^, Luisa Heidemann ^1,2^, Friederike Hesse ^1,5^, Yu Liu ^1,2^, Justus Ramtke ^1,2^, Nicola Beindorff ^6^, Frank Hofheinz ^7^, Avan Kader ^1, 8^, Michael Mülleder ^9^, Kathrin Textoris-Taube ^9^, Bernd Hamm ^1^, Dominik N. Müller ^2,10,11^, Robin Schmidt ^1,2^, Ralf Hauptmann ^1^, Heike Traub ^12^, Mathias Schannor ^12,13^, Lynn Jeanette Savic ^1,2,5,14 *^

1 Department of Radiology, Charité – Universitätsmedizin Berlin, Corporate Member of Freie Universität Berlin and Humboldt‐Universität zu Berlin, Berlin, Germany

2 Experimental and Clinical Research Center (ECRC), A Cooperation of Max Delbrück Center for Molecular Medicine and Charité – Universitätsmedizin Berlin, Berlin, Germany

3 Department of Nuclear Medicine, Campus Virchow-Klinikum, Charité-Universitätsmedizin Berlin, Berlin, Germany

4 Department of Molecular Toxicology, German Institute of Human Nutrition Potsdam-Rehbruecke (DIfE), 14558 Nuthetal, Germany

5 Berlin Institute of Health at Charité – Universitätsmedizin Berlin, Berlin, Germany.

6 Berlin Experimental Radionuclide Imaging Center (BERIC, RRID: SCR_028573), Charité – Universitätsmedizin Berlin, Corporate Member of Freie Universität Berlin and Humboldt‐Universität zu Berlin, Berlin, Germany

7 Helmholtz – Zentrum Dresden – Rossendorf, PET Center, Institute of Radiopharmaceutical Cancer Research, Dresden, Germany

8 Department of Diagnostic and Interventional Radiology, Technical University of Munich, Munich, Germany

9 Core Facility High-Throughput Mass Spectrometry, Charité – Universitätsmedizin Berlin, Freie Universität Berlin and Humboldt – Universität zu Berlin, Berlin, Germany

10 Charité – Universitätsmedizin Berlin, Corporate Member of Freie Universität Berlin and Humboldt – Universität zu Berlin, Berlin, Germany

11 Max Delbrück Center for Molecular Medicine in the Helmholtz Association, Berlin, Germany

12 Bundesanstalt für Materialforschung und -prüfung (BAM), Berlin, Germany

13 Freie Universität Berlin, Institute of Geological Sciences, Berlin, Germany

14 Department of Radiology and Nuclear Medicine, Otto – von – Guericke University Magdeburg, Magdeburg, Germany

‡ These authors contributed equally to this work.

* Corresponding author: lynn-jeanette.savic@charite.de

**Figure S1. Longitudinal body-weight changes across the three experimental groups.** Body weight was monitored throughout the experimental period in the control, short-term, and long-term groups (n = 8 per group). Follow-up duration differed according to the predefined experimental endpoint of each group. Data are presented as mean ± SD. No marked body-weight loss was observed over the study period.

**Figure S2. Longitudinal changes in tumor volume following incomplete microwave ablation (iMWA).** Whole tumor volume was measured on MRI before iMWA and at the corresponding predefined post-iMWA endpoint in the short-term and long-term groups. Each line connects paired measurements from the same rabbit. No inferential statistical comparison was performed.

**Figure S3. Representative dynamic SUV-time curves during the first 10 minutes after [^18^F]FDG injection across the different groups.** The aorta shows a sharp early peak consistent with the arterial input function, while fat exhibits minimal uptake, serving as a low-uptake reference. The liver displays high early FDG uptake and reaches a stable plateau within 10 minutes; in the control group, liver and tumor curves largely overlap during this early phase, indicating perfusion-dominated tracer distribution. In the post-iMWA groups, dynamic SUV curves demonstrate time-dependent differences in tracer distribution between liver and tumor compartments, with relatively greater persistence of [^18^F]FDG activity in tumor tissue, particularly in residual viable tumor regions.

**Figure S4. Quantification of CD45- and CD11b-positive areas across the three experimental groups.** **(A, B)** Representative immunohistochemical staining for CD45 **(A)** and CD11b **(B)** in the control, short-term, and long-term groups at 5×, 10×, and 40× magnification. **(C, D)** Quantification of CD45-positive areas **(C)** and CD11b-positive areas **(D)**, expressed as the percentage of positively stained area. Data are presented as box-and-whisker plots, with the center line indicating the median, the box representing the interquartile range, and the whiskers indicating the minimum and maximum values. Statistical significance was assessed using one-way ANOVA followed by Tukey’s multiple-comparisons test. *p < 0.05, **p < 0.01, ***p < 0.001, ****p < 0.0001.

**Appendix S1**

**1. Rabbit VX2 Liver Tumor Model**

New Zealand white rabbits (male, 2.5-4.2 kg, Charles River Laboratories) were maintained in laminar flow rooms at constant temperature and humidity, with food and water provided ad libitum. VX2 tumor chunks were injected into the quadriceps muscle of the hind leg of donor rabbits (n=6), and tumors were allowed to grow for 4 weeks. The tumor chunks were harvested from the donor animal, and approximately 0.3-0.5 mL was injected into the left liver lobe of a recipient rabbit via median laparotomy using an 18-G catheter. Abdominal fascia and skin were closed in two layers using absorbable suture materials (chromic gut 3-0; Vicryl 4-0). The tumors were allowed to grow for 14 days until a well-delineated solitary tumor (1.5-2 cm in diameter) was measurable on MRI. Additionally, rabbits were randomly assigned to one group, meaning that at the time of tumor implantation, a randomization software assigned them to one of the groups. This way, we avoided any bias (e.g., due to tumor size or appearance based on the baseline MRI), but it still gave us enough time to organize the following imaging and treatment sessions. Surgeries and PET/MRI were performed under general anesthesia using isoflurane 1.5%–3% in oxygen. Heat support was provided to prevent hypothermia, and vital parameters such as oxygen saturation, heart rate, and body temperature were monitored during experimental procedures. Prior to all surgical interventions, analgesic buprenorphine (0.03 mg/kg, CP-Pharma, Germany) and meloxicam (0.3 mg/kg, Boehringer Ingelheim, Ingelheim, Germany) were injected subcutaneously. In addition, meloxicam (0.3 mg/kg) was injected subcutaneously for the following three days after all surgical procedures.

**2. Incomplete Microwave Ablation**

For incomplete microwave ablation (iMWA), a microwave needle (17 F, NeuWave Medical, Inc.) with a 1-2 cm tip was placed in the center of the tumor along the long axis of the tumor. The needle position was stabilized using the device-specific freezing-lock mechanism to prevent displacement. Subsequently, CT was used to confirm the correct placement of the needle. MWA was performed using a microwave generator. The power output was set at 30 W for approximately 40-60 seconds, with the aim of covering at least 50% of the tumor with the ablation zone. The temperature in the ablation zone around the microwave needle was 100-103℃, as indicated by the system. All microwave ablation procedures were performed under direct visualization. The tumor was first exposed after laparotomy, followed by direct insertion of the microwave probe into the tumor and activation of the freezing-lock mechanism under visual guidance. CT imaging was then used to confirm the exact position of the probe tip and active ablation segment. Ablation parameters were set and monitored on the control panel, during which a visible core zone of coagulative necrosis developed within the tumor. Finally, the probe was withdrawn, and the ablation extent was verified. This standardized procedure was used to achieve partial tumor ablation while preserving a viable non-ablated residual tumor region for subsequent imaging and tissue analyses.

**3. Multimodal Imaging Protocol and Quantitative Image Analysis**

For treatment groups (short-term and long-term), baseline scans were acquired 1 day prior to treatment; the control group underwent baseline imaging on the first of three consecutive scanning days. Baseline imaging protocol included localizer, diffusion-weighted imaging (DWI, 2500/51) with ADC maps, T2-weighted sequences (4500-4600/78, both respiratory gated using pacing), T1-weighted imaging (repetition time msec/echo time msec, 5.27/2.46) before and after gadolinium-based contrast injection (Primovist, 0.1 mmol/kg, Bayer, Germany), and dynamic contrast-enhanced sequences (DCE), which were acquired in axial plane every 4 seconds covering the entire liver over approximately 10 minutes.

Experimental imaging was performed over three consecutive scanning days, as described in the manuscript. On Day 2, rabbits received Eu-VSOP (0.05 mmol/kg, Charité-Universitätsmedizin Berlin, Germany; particle size ~7-11 nm, more details regarding its physicochemical characterization are provided in Table S3), which was developed by Angela Ariza de Schellenberger et al. in 2017 ^[1]^, consists of superparamagnetic iron oxide nanoparticles labeled with europium. On Day 3, no MR contrast agent was administered. Rabbits received [^18^F]FDG (FCON, Germany; 80 MBq/rabbit) for PET scanning, including a 10-min dynamic acquisition and a static acquisition at approximately 1 h after injection, to assess tumor viability and glucose metabolism. T2-weighted sequences were also acquired on Day 3. Detailed parameters for multiparametric PET/MRI protocol are shown in Table S1.

For quantitative images, the analysis of tumor segmentation, mean signal intensities, regions of interest (ROIs) and tumor volumetry is described in the “Methods” section of the manuscript.

**4. Tissue Processing and Molecular Analysis**

**4.1 *Tissue harvest and processing-*** Rabbits were euthanized by intravenous injection of Euthasol (1 mL/kg, CP-Pharma Handelsgesellschaft mbH, Germany) immediately at the predefined study endpoint. The tumor, adjacent tissue, and contralateral liver were harvested. All tissues were fixed in formaldehyde and embedded in paraffin for subsequent analyses.

**4.2 *Hematoxylin and eosin staining, Immunohistochemistry, and Prussian blue staining-*** Tissue sections were cut at a thickness of 5 μm. Hematoxylin and eosin (H&E) staining (X903.2 and X883.2, Roth) was performed to confirm residual tumor viability and to provide a histological reference for other ex vivo studies. Standard immunohistochemistry (IHC) staining protocols were used with the following antibodies: anti-HLA-DR (1:50, ab20181, Abcam), anti-CD45 (1:200, PA5-96061, Thermo Fisher), anti-CD11b (1:200, PA5-79532, Thermo Fisher), anti-CD206 (1:200, sc-376108, Santa Cruz), and anti-MMP-9 (1:100, MA5-15886, Thermo Fisher). Goat Anti-Mouse IgG H&L (HRP) (1:200, ab205719, Abcam) was used as secondary antibody. Antibody diluent was obtained from Thermo Fisher (003118). Avidin/Biotin blocking reagents and other reagents were obtained from two kits (ab64212 and ab64264, Abcam) and were used according to the manufacturer’s instructions. Prussian blue staining (131505.121, PanReac AppliChem ITW Reagents) was performed to visualize iron deposition associated with Eu-VSOP in the target tissues. IHC-stained slides were digitized by whole-slide scanning for quantification, followed by anonymization of all digitized slides by a researcher not involved in subsequent analyses, and allocation of code identifiers. The other two researchers responsible for quantitative analysis were blinded to the experimental group, the time point following ablation, and the corresponding imaging results. Using QuPath (version 0.5.0), three non-overlapping regions of interest (ROIs) were delineated for each sample. ROIs were selected based on predefined anatomical and histological criteria, and areas containing major tissue artifacts, folds, or extensive areas of non-neoplastic necrosis were excluded. The size and positioning of the three ROI boxes were kept as consistent as possible across samples. Positivity rates were calculated by averaging the values obtained from the three ROIs.

**4.3 *Laser ablation-inductively coupled plasma time-of-flight mass spectrometry (LA-ICP-ToF-MS) -*** It was performed as described previously ^[2]^ for quantitative elemental imaging of ^153^Eu to determine the Eu-VSOP distribution in the presence of endogenous iron within the peritumoral area after Eu-VSOP administration ^[3]^. The slices (5 μm thick) were deparaffinized and dried overnight. A NWRimage laser ablation system (266 nm, Elemental Scientific Laser, Bozeman, USA) equipped with a low-dispersion ablation cell in a TwoVol3 ablation chamber was attached to an ICP-ToF-MS (Model 2R, Tofwerk AG, Thun, Switzerland) using a 1.016 mm ID PEEK tubing and a Dual Concentric Injector (DCI2). Instrumental settings are summarized in Table S5. The laser system was controlled by the ActiveView2 software (Elemental Scientific Laser, Bozeman, USA). A square laser beam of 20 μm × 20 μm was used to produce high resolution chemical maps. The samples were scanned underneath a pulsed beam with a repetition rate of 100 Hz and a fluence of 10J/cm^2^ and ablation spots were lined up edge-to-edge across each line and between consecutive lines without overlapping areas. Laser ablation in the TwoVol3 ablation chamber was carried out in a helium atmosphere using a combined helium flow of 0.65 L min^-1^ and an additional argon flow of 0.95 L min^-1^ injected at the torch of the ICP-ToF-MS. The injection of H_2_ and He into the collision/reaction cell (CRC) of the ICP-ToF-MS reduces ^40^Ar^+^ and ^40^Ar^40^Ar^+^ interferences ^[4]^. A H2/He gas (8.1 %H_2_ and 91.9 % He) was used with a flow rate of approximately 4.0 mL min^-1^. Instrumental settings were optimized tuning with NIST SRM610 glass focusing on high intensities, low oxide formation rates and low elemental fractionation based on ^238^U^+^/^232^Th^+^ values close to 1. The effective wash-out time was 10 ms (full-width at 1 percent maximum, FW0.01M) based on the signal from ^28^Si of individual pulses on NIST SRM 610 glass and from the ^63^Cu signal of individual pulses on gelatin standards.

Data acquisition was triggered for every ablation position using the laser ablation workflow of TofPilot (Tofwerk AG, Thun, Switzerland) acquiring the entire image as a single Hierachical Data Format (HDF5) file. Gas blank signals were measured before each sample and calibration material, respectively. Data post-processing of the acquired images and reference materials was conducted in two steps. The raw data were first processed with the Tofware software (TOFWERK AG, Thun, Switzerland) to select isotopes, refine peak shapes, perform mass calibration, and model and subtract baseline signal intensities. Afterwards, the modified HDF5 files were loaded in Iolite v4 software (Elemental Scientific Laser, Bozeman, USA) and the data were reduced using the “3D Trace Elements” (3D-TE-DRS) data reduction scheme^[5-7]^. Gelatin multi-element standards (BIO-logi-CAL standard 2, 48 elements; aemas, Oosterhout, The Netherlands) were used as calibration materials following recently established procedures ^[8]^ and analyzed before and after samples.

**4.4 *Proteomics analysis***

Proteomics data were incorporated into the scheme of the liver metabolism model adapted from Berndt et al. to analyze metabolic changes dominated by glucose, lipid, and amino acid metabolism ^[9]^. Details on Liquid Chromatography with tandem mass spectrometry (LC-MS/MS) are provided as follows.

**4.4.1 *Shotgun proteome profiling and data analysis***

*(1) Sample preparatio*n:

The paraffin-embedded tissue was cut into 10-20μm sections, and as much excess paraffin as possible was removed around the tissue. The liver and tumor (ablated, non-ablated) tissues in three groups were placed into test-specific 8-tube strips one by one, with the total weight of each sample to be 1-1.5 mg and 100 µl were added and the volume was made up to 100 µl of TLB (Tissue Lysis Buffer). Samples were deparaffinized and homogenized in a Covaris LE220Rsc with the following settings:

Temperature 20°C, Duty Factor 25%, 300 s, 350 W peak power and plate definition Dithering 1 mm Y-dither @ 20 mm/ s. Debris was collected (2500 rpm for 5 min), protein concentration determined (Pierce Protein Assay Kit, 23225).

Lysates were processed using the single-pot solid-phase-enhanced sample preparation (SP3) protocol with two-step reduction and alkylation. Briefly, 5 μl of reduction buffer (100 mM DTT, Dithiothreitol) was added, then samples were incubated at room temperature for 20 min. For alkylation the samples were incubated under the same conditions with 5µl 400mM CAA (chloroacetamide). To bind the proteins, paramagnetic beads (1:1 ratio hydrophilic/hydrophobic) were used, and the proteins were precipitated by adding to an end concentration of 68 % ACN. Samples were washed five times with isopropanol and once with 100% ACN. 35µl 100mM ABC and 0.2µg/ µl Trypsin (enzyme ratio of 1:100 (w/w)) were added and the lysates were incubated at 37°C with shaking overnight. The reaction was stopped by formic acid to a final concentration of 0.1%. Peptide concentration was determined (Pierce 23290), and 200 ng peptide mixtures were analyzed by LC-MS/MS without further conditioning or clean-up.

*(2) Liquid Chromatography with tandem mass spectrometry analysis:*

Liquid Chromatography with tandem mass spectrometry (LC-MS/MS) was carried out by a SCIEX ZenoTOF 7600 System mass spectrometer, coupled to a Waters ACQUITY UPLC M-Class System. The LC separation was performed using a Waters HSS T3 column (300um x 150mm, 1.8um) heated to 35°C. Briefly, peptides were eluted using a gradient from 3 % to 40 % solvent B over 19 min at 5 µL per minute flow rate. Solvent A was 0.1 % formic acid, and solvent B was 100% and 0.1 % formic acid. Peptides were detected by a ZenoSWATH MS/MS acquisition scheme with 11 ms accumulation time and 85 variable size windows Ion source gas 1 &2 were set as 12 and 60 psi respectively; Curtain gas 55, CAD gas 7 and source temperature at 150°C; As source parameters spray voltage was set at 4500V.

*(3) Protein identification and quantification:*

Raw data were processed with DIA-NN (version 1.8.1) (Demichev et al. 2019) using the default settings with fragment ion m/z range set from 100-1800, mass accuracy set to 20 ppm and 12 ppm at the MS2 and MS1 level, respectively, scan window set to 7, MBR (Match-between-runs) enabled and Quantification strategy set as “Robust LC (high Precision)”. A spectral library free approach and OryctolagusCuniculus UniProt (UP000001811, downloaded on 07.03.2024) were used for annotation. The output was filtered at 1% FDR on peptide level.
**4.4.2 *Assessment of metabolic capacities based on proteomic data***

Hepatic metabolic capacities were assessed using the HEPATOKIN1 model ^[9]^ combined with a detailed representation of hepatic lipid droplet metabolism ^[10]^. This integrated approach covered the key metabolic pathways involved in carbohydrate metabolism, lipid metabolism, and amino acid metabolism within hepatocytes, as first described in ^[9]^. Hepatic energy metabolism considering electrophysiological processes at the inner mitochondrial membrane, including the generation and use of the proton motive force, was modeled using kinetic equations based on the Goldman-Hodgkin-Katz framework ^[11]^. Hormone-dependent regulation of liver metabolism through reversible enzyme phosphorylation was incorporated using a phenomenological transfer function, as detailed in ^[12]^. Proteomic profiles were used to create individualized model instances following the methodology described in ^[9, 13]^.

Individual model instantiations were generated using protein intensity profiles obtained from quantitative shotgun proteomics to scale the maximal activities of enzymes and transporters. This approach is based on the principle that the maximal activity of an enzyme is proportional to its protein abundance, described by the equation:

$$v_{max}^{sample}=v_{mx}^{mean control}\frac{E^{sample}}{E^{mean control}}$$

where $v_{max}^{mean control}$represents the maximal activity in control tissue, obtained from ^[9]^; $E^{mean control}$ denotes the mean protein abundance in the control group; and $E^{sample}$ denotes the protein abundance of enzyme E in the sample..

Metabolic physiological functions were defined as the maximal fluxes under specified physiological conditions, particularly within a plasma glucose concentration range of 3–12 mM. All simulations were performed using MATLAB Release 2023b.

**4.4.3 *Statistical analysis***

Statistical analysis, cluster analysis, and Pearson correlation analysis were performed using MATLAB Release 2023b with the bioinformatics toolbox (The MathWorks, Inc., Natick, MA, USA). Cluster analysis was performed using the clustergram function. The values of the calculated metabolic functions were transformed to mean “0” and standard deviation “1”. Group values were checked for normality by a one-sample Kolmogorov-Smirnov test. Statistical group differences were calculated by the unpaired two-sided Student’s t-test for normally distributed group values, otherwise, the Wilcoxon signed-rank test was used.

**4.4.4 *Protein-level gene set enrichment analysis of LC–MS/MS data***

Gene set enrichment analysis (GSEA) was conducted using gene sets from the Molecular Signatures Database (MSigDB) ^[14, 15]^. The analysis compared liver and tumor (untreated, treated) samples from three groups. The false discovery rate (FDR) was estimated using a gene set-based permutation test with 1,000 permutations. Due to the small sample size (n = 40), only pathways with an FDR/q-value ≤ 0.25 were included in the analysis. Pathways with an FDR/q-value ≤ 0.05 were considered significantly enriched.

**4.4.5 *Macrophage polarization- and immunosuppression-related signature analysis based on proteomic data***

We assessed pathway level functional activity linked to macrophage phenotypic shifts and immune suppressive responses from VX2 liver proteome profiles. A curated signature aggregation workflow (custom multi-gene Stouffer-type framework) was deployed to compute functional activity metrics for 9 distinct biological pathways. Only quantified proteins bearing valid fold change estimates and statistical test outputs were retained for downstream signature aggregation. Manually curated signature panels (6 genes per biological theme) capturing macrophage activation and polarization, immune checkpoint function, myeloid derived suppressor cell features and other ablation associated immune programs were applied to the dataset (Table S6). Per protein nominal P values were transformed into two sided Z statistics, whose algebraic sign was assigned according to log₂ transformed fold change values to reflect up or down regulation. Genes belonging to predefined signatures yet undetected within the measured proteome were discarded before aggregation. Composite pathway activity metrics were generated by summing all signed Z values from detectable signature components, normalized against the square root count of successfully quantified signature members. Resulting composite Z metrics signify functional pathway status: positive values indicate functional activation, while negative values denote pathway repression. Computed composite activity scores corresponding to D3 and D14 experimental groups were visualized within a two-dimensional heatmap layout.

Protein interaction networks were assembled and inspected via the STRING database (version 12.5; https://string db.org/). Inter protein connections were incorporated using a medium confidence interaction threshold set at 0.400. To resolve discrete functionally coordinated protein communities embedded within the global interactome, Markov Cluster Algorithm (MCL) clustering was performed with an inflation parameter of 3, which favors partitioning into fine scale functional modules. Only clusters encompassing ≥3 distinct protein coding entries were kept for further interpretation. Functional theme over representation analysis was subsequently carried out to interpret biological properties of recovered network modules. Gene constituents for every MCL derived cluster are documented in Table S7.

**Table S1: Imaging Parameters for Multiparametric PET/MRI Protocol**

| **Sequence** | **Plane** | **TR (ms)** | **TE (ms)** | **Flip Angle (°)** | **FOV (mm²)** | **Matrix** | **Slice Thickness (mm)** | **Slice Gap (mm)** | **Other Parameters** |
| --- | --- | --- | --- | --- | --- | --- | --- | --- | --- |
| **T1-weighted VIBE Dixon** | Axial | 5.27 | 2.46 | 9 | 118 × 199 | 228 × 384 | 2.5 | N/A | Fat/water separation; pre-/post-contrast |
| **T1-weighted DCE** | Axial | 3.45 | 1.28 | 9 | 118 × 199 | 63 × 192 | 2.5 | N/A | Temporal resolution: ~3–5 s/frame; Primovist |
| **T2-weighted TSE** | Axial | ≈4500 4600 | 78 | 160 | 131 × 200 | 118 × 256 | 3.0 | 0.0 | Fat suppression; high tissue contrast |
| **DWI (b = 50, 400, 800)** | Axial | 2500 | 51 | 90 | 160 × 199 | 90 × 112 | 3.0 | 0.5 | EPI readout: ADC maps calculated |
| **[^18^F]FDG PET** | Axial | - | - | - | 718 × 718 | 344 × 344 | 2.03 | 0.0 | Static scan at 60 min p.i.; 10 min dynamic acquisition |

**Table S2: Representative T2-TSE Sequence Parameters Relevant to Eu-VSOP-Associated Susceptibility Effects**

| **Parameter (T2-TSE)** | **Pre-Eu-VSOP injection** | **24 h Post-Eu-VSOP injection** |
| --- | --- | --- |
| Echo spacing (ms) | 4.8 | 4.8 |
| Turbo factor (ETL) | 119 | 119 |
| Bandwidth (Hz/Px) | 490 | 490 |
| TE (ms) | 80 | 80 |
| Gradient mode | Normal | Normal |
| Parallel imaging (PAT) | GRAPPA 3 | GRAPPA 3 |
| Refocusing flip angle | Refocusing Flip Angle <180° | Refocusing Flip Angle <180° |
| Slice thickness (mm) | Showed in Table S1 | Showed in Table S1 |

**Table S3: Key Physicochemical Characterization of Eu-VSOP nanoparticles^[1]^**

| **Parameter** | **Value** |
| --- | --- |
| Eu:Fe mass ratio | 0.0274 (≈ 2.74 % Eu relative to Fe) |
| Core size (TEM) | ≈ 5–7 nm |
| Hydrodynamic diameter (DLS) | 9.9 ± 2.1 nm |
| Zeta potential (pH ≈ 7) | –25 to –30 mV (similar to VSOP) |
| Magnetization (Mₛ) | 69.6 (Am²/kg Fe)⁻¹ |
| Relaxivities (37°C, 20–40 MHz) | r₁ = 19 ; r₂ = 53 (L/mmol s) |
| Colloidal stability | Stable > 2 years |
| Fluorescence properties | λₑₓ ≈ 338 nm; λₑₘ ≈ 616 nm |

**Table S4: Inter-reader Agreement using Intraclass Correlation Coefficients (ICC) across four ROIs among three groups.**

| **ROI Parameter** | **Timepoint** | **N** | **ICC(2,1)** | **ICC(2,2)** | **95% CI** | **Agreement** |
| --- | --- | --- | --- | --- | --- | --- |
| Peritumoral Thickness | Control | 8 | 0.936 | 0.951 | 0.773 - 0.975 | Excellent |
|  | Short-term | 8 | 0.512 | 0.544 | -0.257 - 0.754 | Moderate |
|  | Long-term | 7 | 0.769 | 0.824 | 0.187 - 0.879 | Good |
|  | Overall | 23 | 0.922 | 0.939 | 0.837 - 0.959 | Excellent |
| Tumor ROI (Total) | Control | 8 | 0.920 | 0.940 | 0.810 - 0.970 | Excellent |
|  | Short-term | 8 | 0.680 | 0.740 | 0.300 - 0.870 | Moderate - Good |
|  | Long-term | 8 | 0.850 | 0.900 | 0.600 - 0.960 | Good - Excellent |
|  | Overall | 24 | 0.890 | 0.940 | 0.880 - 0.970 | Excellent |
| Treated Tumor ROI | Short-term | 8 | 0.600 | 0.650 | 0.100 - 0.820 | Moderate |
|  | Long-term | 8 | 0.860 | 0.910 | 0.620 - 0.960 | Good - Excellent |
|  | Overall | 16 | 0.860 | 0.910 | 0.840 - 0.970 | Excellent |
| Untreated Tumor ROI | Control | 8 | 0.934 | 0.960 | 0.860 - 0.976 | Excellent |
|  | Short-term | 8 | 0.601 | 0.646 | 0.501 - 0.805 | Moderate |
|  | Long-term | 8 | 0.851 | 0.893 | 0.607 - 0.959 | Good - Excellent |
|  | Overall | 24 | 0.910 | 0.952 | 0.781 - 0.950 | Good - Excellent |

**Table S5: Instrument Settings and Data Acquisition Conditions for LA-ICP-ToF-MS Imaging**

| NWRimage Laser Ablation system settings | |
| --- | --- |
| Fluence | 10 J cm^-2^ |
| Repetition rate | 100 Hz |
| Beam size | 20 µm |
| Mask shape | Square |
| Scan velocity | 2000 µm s^-1^ |
| He chamber gas flow | 0.30 L min^-1^ |
| He cup gas flow | 0.35 L min^-1^ |
| Wash-out time (FW0.01M) | 10 ms |
| icpToF 2R settings |  |
| RF power | 1550 W |
| Ar cool flow | 14 L min^-1^ |
| Ar auxiliary flow | 0.8 L min^-1^ |
| Ar nebulizer flow | 0.95 L min^-1^ |
| Sampling depth | 4 mm |
| CRC H_2_/He gas flow | 4 mL min^-1^ |

**Table S6: Curated Gene Sets used for Stouffer-based Pathway Scoring**

| **Process** | Gene 1 | Gene 2 | Gene 3 | Gene 4 | Gene 5 | Gene 6 |
| --- | --- | --- | --- | --- | --- | --- |
| **Immune checkpoints tolerance** | LGALS3 | LGALS1 | CD200 | STC1 | ENTPD1 | - |
| **Myeloidderived suppressor cell signature** | S100A9 | S100A8 | LCN2 | MMP | SPP1 | GPD2 |
| **Adenosinergic Immune Suppression** | ENTPD1 | NT5E | PDE4D | NT5DC2 | - | - |
| **T Cell Activation (TCR signaling)** | CD3E | CD2 | LCK | PTPRC | VAV1 | FYN |
| **Apoptosis of Immune Cells** | CASP3 | CASP7 | CASP9 | BAX | BAK1 | - |
| **Macrophage Activation** | FCGR3B | LYZ | CSF1R | CD14 | TOP3A | CD68 |
| **Monocyte-to-Macrophage Transition** | CD14 | CD68 | CSF1R | ITGAM | FCGR1A | FCGR3B |
| **M1 Macrophage activation** | TNFRSF1A | IL1B | CSF1R | CAT | GPX1 | CD36 |
| **TGF‑beta/SMAD Signaling M2** | TGFBR1 | TGFBR2 | SMAD2 | SMAD3 | SMAD4 | IRF4 |

**Table S7: Protein membership of MCL derived functional clusters from STRING protein interaction network**

| **cluster number** | **cluster color** | **gene count** | **primary description** | **secondary description** | **protein names** |
| --- | --- | --- | --- | --- | --- |
| **1** | Red | 11 | Immunosuppressive M2‑like macrophage  regulatory module | TGF‑β signaling & inhibitory immune receptors | LAG3, CD38, CD44, SPP1,  STAT6, IRF4, CD200, ITGAL, CD300LF, TGFBR1, TGFBI |
| **2** | Brown | 8 | Scavenger receptor Cys-rich | Homeostatic clearance by myeloid cells | CD163, CD68, CLEC7A, CD14,  MERTK, HMOX1, MSR1, IL1R2 |
| **3** | Dark Golden Rod | 5 | PI3K‑AKT‑MAPK pro‑tumour signalling module | Intracellular oncogenic signal transduction | GSK3B, AKT3, MAPK1, PIK3CA, PIK3CD |
| **4** | Green | 4 | MHC‑I antigen processing complex | TAP complex | PSMB8, TAP2, TAP1, PSMB10 |
| **5** | Green 2 | 4 | Glycolysis / fructose‑6‑phosphate metabolism | Myeloid cell metabolic reprogramming | PKM, PFKP, HK1, HK2 |
| **6** | Cyan | 4 | Galactoside-binding lectin | - | LGALS3, GPNMB, LGALS3BP,  LGALS1 |
| **7** | Dark Cyan | 3 | MHC‑II exogenous antigen presentation | Antigen processing for myeloid‑mediated T‑cell priming | CD74, HLA-DQB2, HLA-DMA |

**5. References**

1.	A. A. de Schellenberger, R. Hauptmann, J. M. Millward, et al., “Synthesis of europium-doped VSOP, customized enhancer solution and improved microscopy fluorescence methodology for unambiguous histological detection,” Journal of nanobiotechnology 15, no. 1 (2017): 71. <https://doi.org/> 10.1186/s12951-017-0301-6

2.	M. Schannor, M. Oelze, H. Traub, et al., “Advancing Biomarker Research: In Situ Cu Isotope Analysis in Liver Tumors by LA-MC-ICP-MS,” Analytical chemistry 97, no. 8 (2025): 4425-32. <https://doi.org/> 10.1021/acs.analchem.4c05626

3.	C. Scharlach, L. Müller, S. Wagner, et al., “LA-ICP-MS Allows Quantitative Microscopy of Europium-Doped Iron Oxide Nanoparticles and is a Possible Alternative to Ambiguous Prussian Blue Iron Staining,” Journal of biomedical nanotechnology 12, no. 5 (2016): 1001-10. <https://doi.org/> 10.1166/jbn.2016.2230

4.	M. Burger, L. Hendriks, J. Kaeslin, A. Gundlach-Graham, B. Hattendorf, D. Günther, “Characterization of inductively coupled plasma time-of-flight mass spectrometry in combination with collision/reaction cell technology – insights from highly time-resolved measurements,” Journal of Analytical Atomic Spectrometry 34, no. 1 (2019): 135-46. <https://doi.org/> 10.1039/C8JA00275D

5.	C. Paton, J. Hellstrom, B. Paul, J. Woodhead, J. Hergt, “Iolite: Freeware for the visualisation and processing of mass spectrometric data,” Journal of Analytical Atomic Spectrometry 26, no. 12 (2011): 2508-18. <https://doi.org/> 10.1039/C1JA10172B

6.	B. Paul, J. Petrus, D. Savard, et al., “Time resolved trace element calibration strategies for LA-ICP-MS,” Journal of Analytical Atomic Spectrometry 38, no. 10 (2023): 1995-2006. <https://doi.org/> 10.1039/D3JA00037K

7.	D. Savard, S. Dare, L. P. Bédard, S.-J. Barnes, “A New Mapping Protocol for Laser Ablation (with Fast-Funnel) Coupled to a Time-of-Flight Mass Spectrometer (LA-FF-ICP-ToF-MS) for the Rapid, Simultaneous Quantification of Multiple Minerals,” 47, no. 2 (2023): 243-65. <https://doi.org/> <https://doi.org/10.1111/ggr.12482>

8.	A. Schweikert, S. Theiner, D. Wernitznig, et al., “Micro-droplet-based calibration for quantitative elemental bioimaging by LA-ICPMS,” Analytical and bioanalytical chemistry 414, no. 1 (2022): 485-95. <https://doi.org/> 10.1007/s00216-021-03357-w

9.	N. Berndt, S. Bulik, I. Wallach, et al., “HEPATOKIN1 is a biochemistry-based model of liver metabolism for applications in medicine and pharmacology,” Nature communications 9, no. 1 (2018): 2386. <https://doi.org/> 10.1038/s41467-018-04720-9

10.	C. Wallstab, D. Eleftheriadou, T. Schulz, et al., “A unifying mathematical model of lipid droplet metabolism reveals key molecular players in the development of hepatic steatosis,” The FEBS journal 284, no. 19 (2017): 3245-61. <https://doi.org/> 10.1111/febs.14189

11.	N. Berndt, O. Kann, H. G. Holzhütter, “Physiology-based kinetic modeling of neuronal energy metabolism unravels the molecular basis of NAD(P)H fluorescence transients,” Journal of cerebral blood flow and metabolism : official journal of the International Society of Cerebral Blood Flow and Metabolism 35, no. 9 (2015): 1494-506. <https://doi.org/> 10.1038/jcbfm.2015.70

12.	S. Bulik, H. G. Holzhütter, N. Berndt, “The relative importance of kinetic mechanisms and variable enzyme abundances for the regulation of hepatic glucose metabolism--insights from mathematical modeling,” BMC biology 14, no. (2016): 15. <https://doi.org/> 10.1186/s12915-016-0237-6

13.	N. Berndt, J. Eckstein, N. Heucke, et al., “Characterization of Lipid and Lipid Droplet Metabolism in Human HCC,” Cells 8, no. 5 (2019): <https://doi.org/> 10.3390/cells8050512

14.	A. Liberzon, A. Subramanian, R. Pinchback, H. Thorvaldsdóttir, P. Tamayo, J. P. Mesirov, “Molecular signatures database (MSigDB) 3.0,” Bioinformatics (Oxford, England) 27, no. 12 (2011): 1739-40. <https://doi.org/> 10.1093/bioinformatics/btr260

15.	A. Subramanian, P. Tamayo, V. K. Mootha, et al., “Gene set enrichment analysis: a knowledge-based approach for interpreting genome-wide expression profiles,” Proceedings of the National Academy of Sciences of the United States of America 102, no. 43 (2005): 15545-50. <https://doi.org/> 10.1073/pnas.0506580102
